# Nitazoxanide inhibits paramyxovirus replication by targeting the Fusion protein folding: role of glycoprotein-specific thiol oxidoreductase ERp57

**DOI:** 10.1038/s41598-018-28172-9

**Published:** 2018-07-11

**Authors:** Sara Piacentini, Simone La Frazia, Anna Riccio, Jens Z. Pedersen, Alessandra Topai, Orazio Nicolotti, Jean-Francois Rossignol, M. Gabriella Santoro

**Affiliations:** 1 Department of Biology, University of Rome Tor Vergata, Rome, Italy; 2 Colosseum Combinatorial Chemistry Centre for Technology, University of Rome Tor Vergata Science Park, Rome, Italy; 3 Department of Pharmaceutical Sciences, University of Bari Aldo Moro, Bari, Italy; 4 Division of Infectious Diseases and International Medicine, University of South Florida College of Medicine, Tampa, Florida USA; 5 Institute of Translational Pharmacology, CNR, Rome, Italy

## Abstract

*Paramyxoviridae*, a large family of enveloped viruses harboring a nonsegmented negative-sense RNA genome, include important human pathogens as measles, mumps, respiratory syncytial virus (RSV), parainfluenza viruses, and henipaviruses, which cause some of the deadliest emerging zoonoses. There is no effective antiviral chemotherapy for most of these pathogens. Paramyxoviruses evolved a sophisticated membrane-fusion machine consisting of receptor-binding proteins and the fusion F-protein, critical for virus infectivity. Herein we identify the antiprotozoal/antimicrobial nitazoxanide as a potential anti-paramyxovirus drug targeting the F-protein. We show that nitazoxanide and its circulating-metabolite tizoxanide act at post-entry level by provoking Sendai virus and RSV F-protein aggregate formation, halting F-trafficking to the host plasma membrane. F-protein folding depends on ER-resident glycoprotein-specific thiol-oxidoreductase ERp57 for correct disulfide-bond architecture. We found that tizoxanide behaves as an ERp57 non-competitive inhibitor; the putative drug binding-site was located at the ERp57-b/b′ non-catalytic domains interface. ERp57-silencing mimicked thiazolide-induced F-protein alterations, suggesting an important role of this foldase in thiazolides anti-paramyxovirus activity. Nitazoxanide is used in the clinic as a safe and effective antiprotozoal/antimicrobial drug; its antiviral activity was shown in patients infected with hepatitis-C virus, rotavirus and influenza viruses. Our results now suggest that nitazoxanide may be effective also against paramyxovirus infection.

## Introduction

The *Paramyxoviridae* form an increasingly diverse family of old and emerging viruses responsible for a wide range of animal and human diseases, including the causative agents of mumps, measles and several respiratory tract infections due to parainfluenza viruses, metapneumoviruses, respiratory syncytial virus (RSV), as well as pathogens responsible for some of the deadliest emerging zoonoses, such as Hendra and Nipah viruses^1,2^.

Based on sequence homology and protein functions, the family is divided into two major sub-families, *Pneumovirinae* and *Paramyxovirinae*, comprising enveloped viruses harboring a nonsegmented negative-sense RNA genome. The plasma membrane-derived lipid envelope contains a sophisticated membrane-fusion machine consisting of the receptor-binding protein (variably called HN/H/G), and the trimeric F-protein, a member of class-I viral fusion glycoproteins^1–4^. The prototype paramyxovirus Sendai (SeV) has been widely used as a model-system for examining the molecular mechanisms that regulate the *Paramyxoviridae* replication cycle. SeV 15,384-bases genome is associated with the nucleocapsid-N protein forming a helical ribonucleoprotein structure (RNP) that serves as template for mRNA transcription and genome replication. The genome encodes the N, P, M, F, HN and L proteins in 6 major open reading frames and the C, V and W proteins by way of overlapping open reading frames and RNA editing^1^. Upon entry into the cell by F-mediated fusion of the viral envelope with the cell membrane, SeV primary mRNAs are synthesized in the host-cell cytoplasm by the virus-associated RNA-dependent RNA-polymerase complex; transcription follows the “stop-start” model^1^. As viral proteins begin to accumulate, viral genomes are replicated via the synthesis of an antigenome N:RNA intermediate and used for secondary transcription. Newly synthesized viral proteins and RNPs assemble together at infected host-cell plasma membranes in preparation for particle budding, which completes the cycle. HN and F glycoproteins sorting to plasma membranes is essential for mature particle formation.

*Paramyxoviridae* F-protein is a type-I transmembrane glycoprotein synthesized as a fusogenically-inactive F_0_-precursor that assembles into a metastable inactive homotrimer known as the prefusion form; F_0_ acquires fusion activity, following cellular protease-mediated cleavage into disulfide-linked chains F_2_ and F_1_. During its synthesis and maturation in the endoplasmic reticulum (ER), following N-glycosylation F-protein interacts with ER molecular chaperones, including lectin chaperones calnexin and calreticulin, and their associated co-chaperone ERp57, a glycoprotein-specific thiol-disulfide oxidoreductase^5–7^. F-protein folding largely depends on ERp57 for the correct dynamics of disulfide-bond formation^5,6^. The correctly folded protein is terminally glycosylated in the Golgi and then transported to the host-cell plasma membrane where it is essential for mature particle formation and mediates fusion between the infected cell and adjacent cells to cause syncytium formation.

Nitazoxanide (NTZ), a thiazolide used in the clinic for treatment of infectious gastroenteritis^8^, and second-generation thiazolides have emerged as a new class of broad-spectrum antiviral drugs^9^. We have previously reported that NTZ and its active circulating-metabolite tizoxanide (TIZ) are effective against influenza virus, hepatitis C and rotavirus infection *in-vitro* as well as in clinical studies^10–14^. Rather than affecting viral targets, thiazolides were suggested to act through a cell-mediated mechanism^10,13^; however, the host target/targets involved in NTZ antiviral activity were not identified as yet.

Herein we investigated the antiviral activity of thiazolides against paramyxovirus infection and explored the molecular mechanism involved, using SeV as a model. The results reveal that NTZ inhibits host ERp57 activity, causing newly synthesized F-protein misfolding, F-aggregate formation and halting F-trafficking to the host plasma membrane.

## Results and Discussion

**Figure 1:**
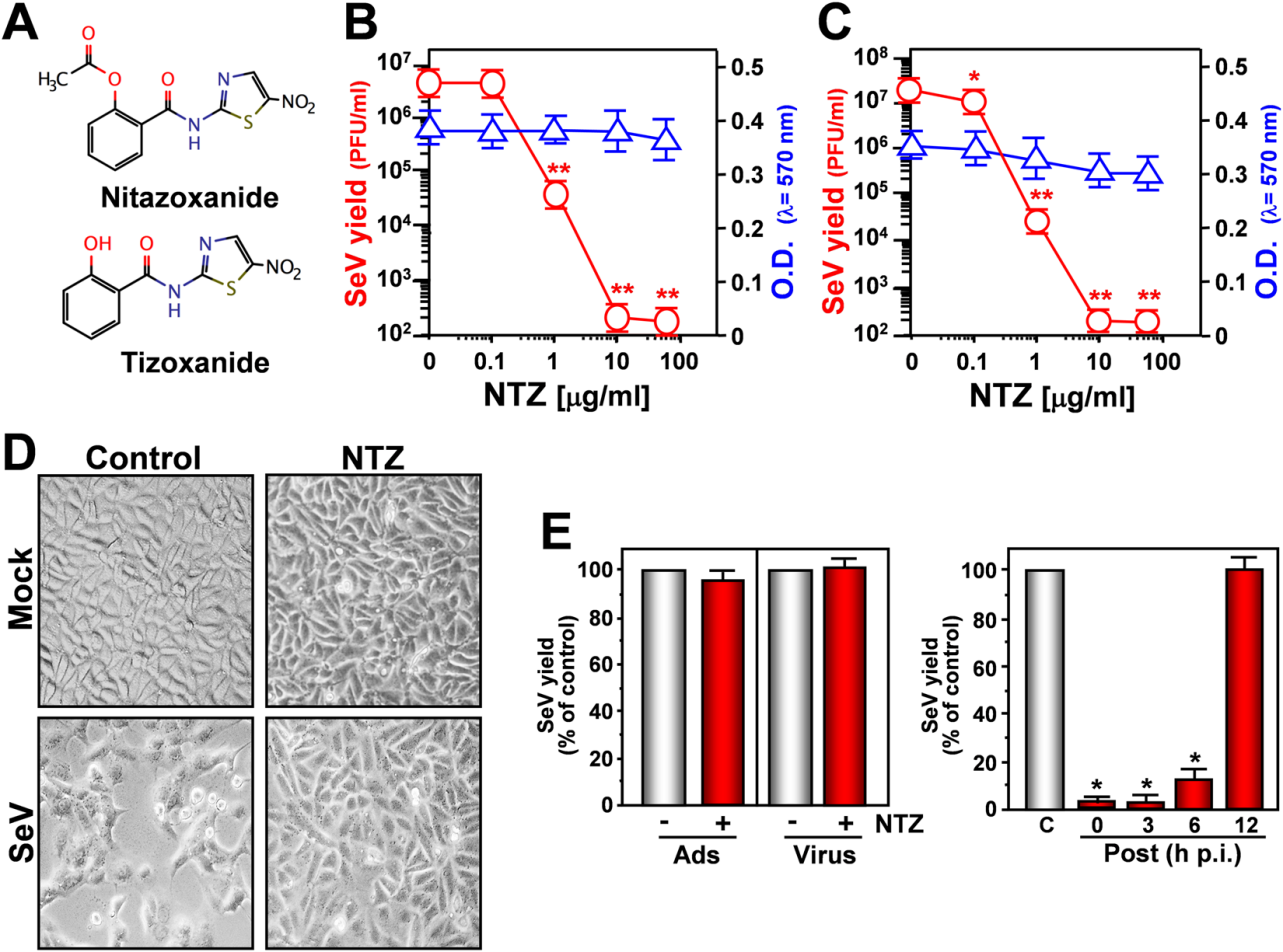
Nitazoxanide inhibits Sendai virus (SeV) replication at post-entry level. (**A**) Structure of nitazoxanide (NTZ) and tizoxanide (TIZ). (**B**,**C**) AGMK cells mock-infected or infected with SeV under single-step (3 PFU/cell) (**B**) and multistep (0.01 PFU/cell) (**C**) conditions were treated with different concentrations of NTZ or vehicle immediately after the adsorption period. Virus yield () was determined at 24 h (**B**) or 48 h (**C**) p.i. by infectivity assay. Data, expressed as PFU/ml, represent the mean ± S.D. of quadruplicate samples. *P < 0.05; **P < 0.01. Cell viability of mock-infected cells () at 24 h (**B**) or 48 h (**C**) was determined by MTT assay (n = 4). Absorbance (O.D.) of converted dye was measured at λ = 570 nm. (**D**) NTZ (10 μg/ml)-induced cytoprotection in AGMK cells infected with SeV (3 PFU/cell) for 24 h (100× magnification). (**E**) SeV-infected AGMK cells were treated with 10 μg/ml NTZ (red bars) or vehicle (gray bars) only during the adsorption period (*Ads*) or at the indicated times after virus adsorption (*Post*); *Virus* = viral inoculum pre-treatment. Virus yield was determined at 24 h p.i. by hemagglutinin titration and expressed as percentage of untreated control (n = 3). *P < 0.01.

Nitazoxanide (Fig. 1A) antiviral activity was initially investigated in monkey kidney (AGMK) cells infected with Sendai virus (SeV) under single-step or multistep conditions. NTZ showed a remarkable antiviral activity against SeV, reducing virus yield dose-dependently with IC_50_ (50% inhibitory concentration) values in the submicromolar range and selectivity indexes ranging from >167 to 625 depending on the multiplicity of infection (MOI) (Fig. 1B,C; Supplementary Fig. 1A,C). Similar results were obtained with the NTZ bioactive metabolite tizoxanide (Supplementary Fig. 1B,C), and several second-generation thiazolides (Supplementary Table 1). Thiazolide antiviral activity was independent of the cell type, being equally effective also in SeV-infected human lung A549 cells (Supplementary Fig. 2A,B). NTZ inhibited SeV replication at concentrations non-toxic for host cells (Fig. 1B,C; Supplementary Fig. 1A and 2A), and was actually cytoprotective in infected cells: SeV-infection is generally characterized by a massive cytopathic effect, causing cell shape and size changes, and nuclear damage, an effect attenuated by NTZ treatment up to 24 h post-infection (p.i.) (Fig. 1D).

NTZ acts at post-entry level, as evidentiated by: *i)* treatment during virus-adsorption or viral inoculum pre-treatment did not inhibit virus replication; *ii*) NTZ-treatment initiated between 0 and 3 h p.i. was equally effective in inhibiting virus replication; *iii*) treatment started as late as 6 h p.i. still reduced viral yield (Fig. 1E), suggesting that the drug is not directly affecting virus binding or entry into target cells, or an early event in the virus replication cycle.

**Figure 2:**
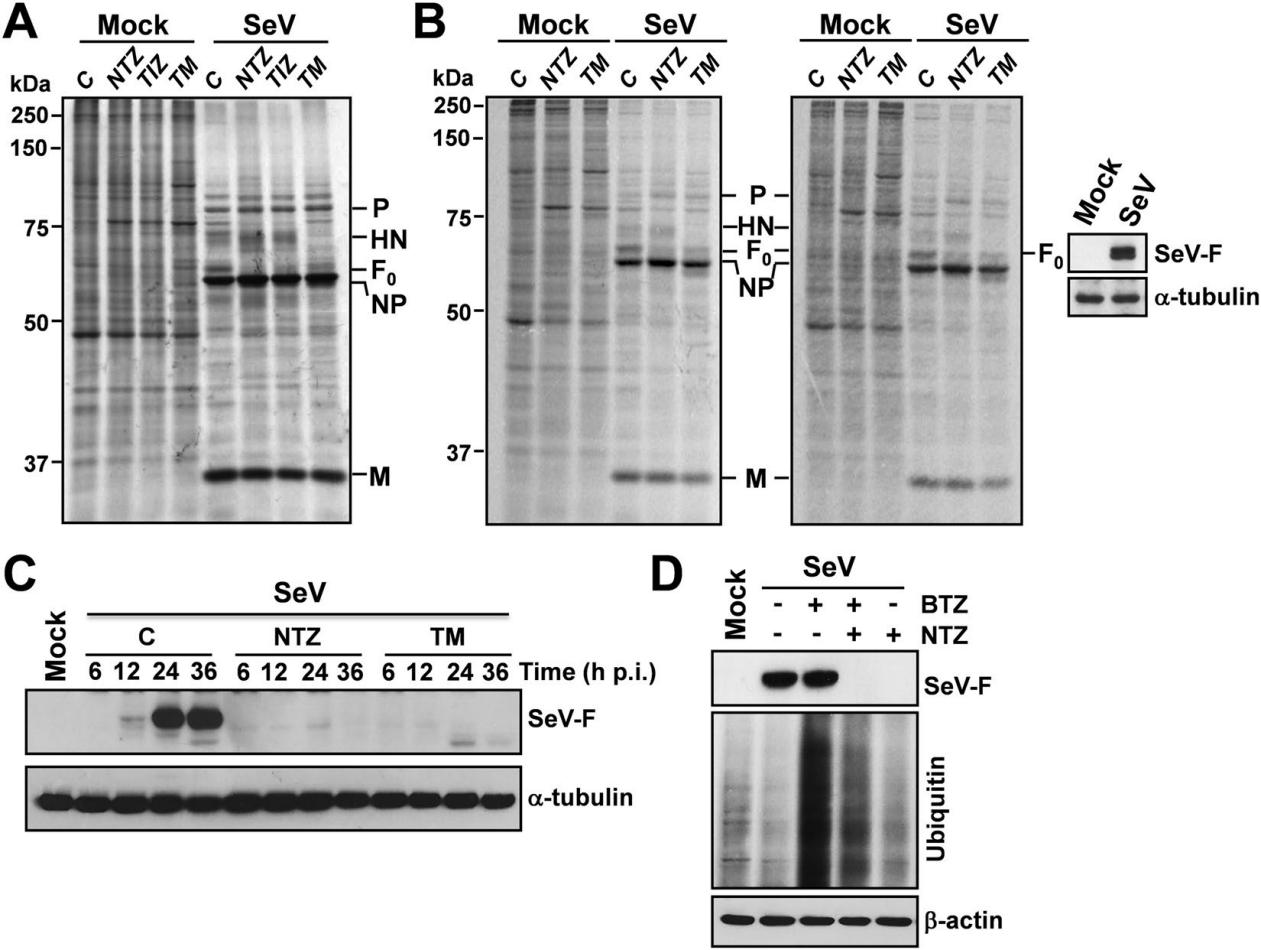
Effect of nitazoxanide on SeV protein synthesis. (**A**,**B**) Autoradiography of [^35^S]Met/Cys-labeled proteins (20 h-pulse; 4-24 h p.i., **A**) (1h-pulse; 23-24 h p.i., **B**) at 24 h p.i. from mock-infected or SeV-infected (3 PFU/cell) AGMK cells treated with 10 μg/ml NTZ, 10 μg/ml TIZ, 2.5 μg/ml tunicamycin (TM) or vehicle (C) after virus adsorption. Samples containing equal amount of radioactivity (**A** and **B**, left panel) or lysate (**B**, right panel) are shown. SeV proteins P (phosphoprotein), HN (hemagglutinin-neuraminidase), F_0_ (fusion glycoprotein F_0_ precursor), NP (nucleoprotein) and M (matrix) are indicated. Identification of SeV-F protein by immunoblot analysis (IB) in duplicate unlabeled mock-infected or SeV-infected samples is shown (**B**). (**C**) IB of SeV-F and α-tubulin levels at different times p.i. in samples treated as in (**B**). (**D**) IB for SeV-F, ubiquitin and β-actin in whole-cell extracts of mock-infected and SeV-infected AGMK cells treated for 24 h with NTZ (10 μg/ml), proteasome inhibitor bortezomib (BTZ) (25 nM) or vehicle. A moderate decrease in ubiquitinated protein levels is noted in bortezomib-treated cells in the presence of NTZ. (**B–D**) Full-length blots/gels are presented in Supplementary Figs 10 and 11.

We have previously shown that, in the case of influenza viruses, thiazolides act by selectively blocking viral hemagglutinin maturation^10^. To investigate whether thiazolides affect parainfluenza protein synthesis/maturation, SeV protein synthesis was analyzed by autoradiography after [^35^S]-methionine/cysteine-labeling and Western-blot in AGMK cells treated with NTZ, TIZ or glycosylation-inhibitor tunicamycin. As shown in Fig. 2A,B, no major changes in SeV protein synthesis were detected at 24 h p.i. after both long (A) and short (B) [^35^S]-methionine/cysteine-labeling pulses in NTZ-treated cells, with the exception of the disappearance of a 66-kDa band, identified as the fusion-protein precursor F_0_ (Fig. 2B,C). NTZ-treatment also caused a transient partial inhibition of protein synthesis in mock-infected cells.

**Figure 3:**
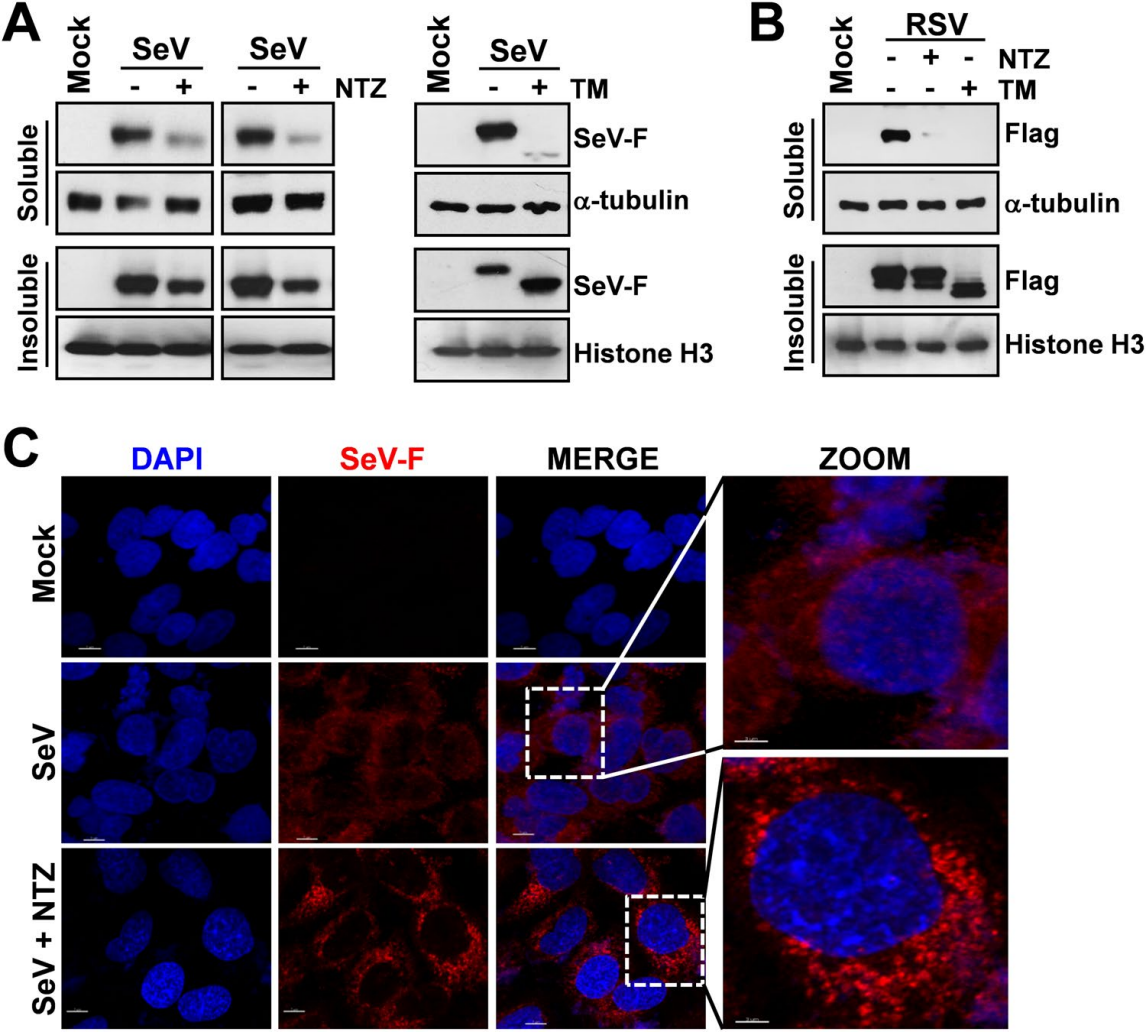
Nitazoxanide causes F-protein insolubilization and aggregate formation. **(A)** IB for SeV-F, α-tubulin and histone-H3 in soluble and insoluble fractions of whole-cell extracts (WCEs) from mock-infected (Mock) or SeV-infected AGMK cells treated for 24 h with NTZ (5 μg/ml), 2.5 μg/ml tunicamycin (TM) or vehicle. Soluble and insoluble fractions from two separate experiments were processed as described in Methods. (**B**) IB for Flag-RSV-F, α-tubulin and histone-H3 in soluble and insoluble fractions of WCEs from HeLa cells transfected with RSV-F/ORF/C-Flag construct (RSV) or pcDNA3 empty vector (Mock), and treated for 20 h with NTZ (5 μg/ml), TM (2.5 μg/ml) or vehicle. (**C**) Confocal images of SeV-F (red) in mock-infected and SeV-infected A549 cells treated with NTZ (10 μg/ml) or vehicle for 24 h. Nuclei are stained with DAPI (blue). Merge and zoom images are shown. Scale bar, 7 μm (zoom, 3 μm). (**A**,**B**) Full-length blots/gels are presented in Supplementary Figs 12 and 13.

**Figure 4:**
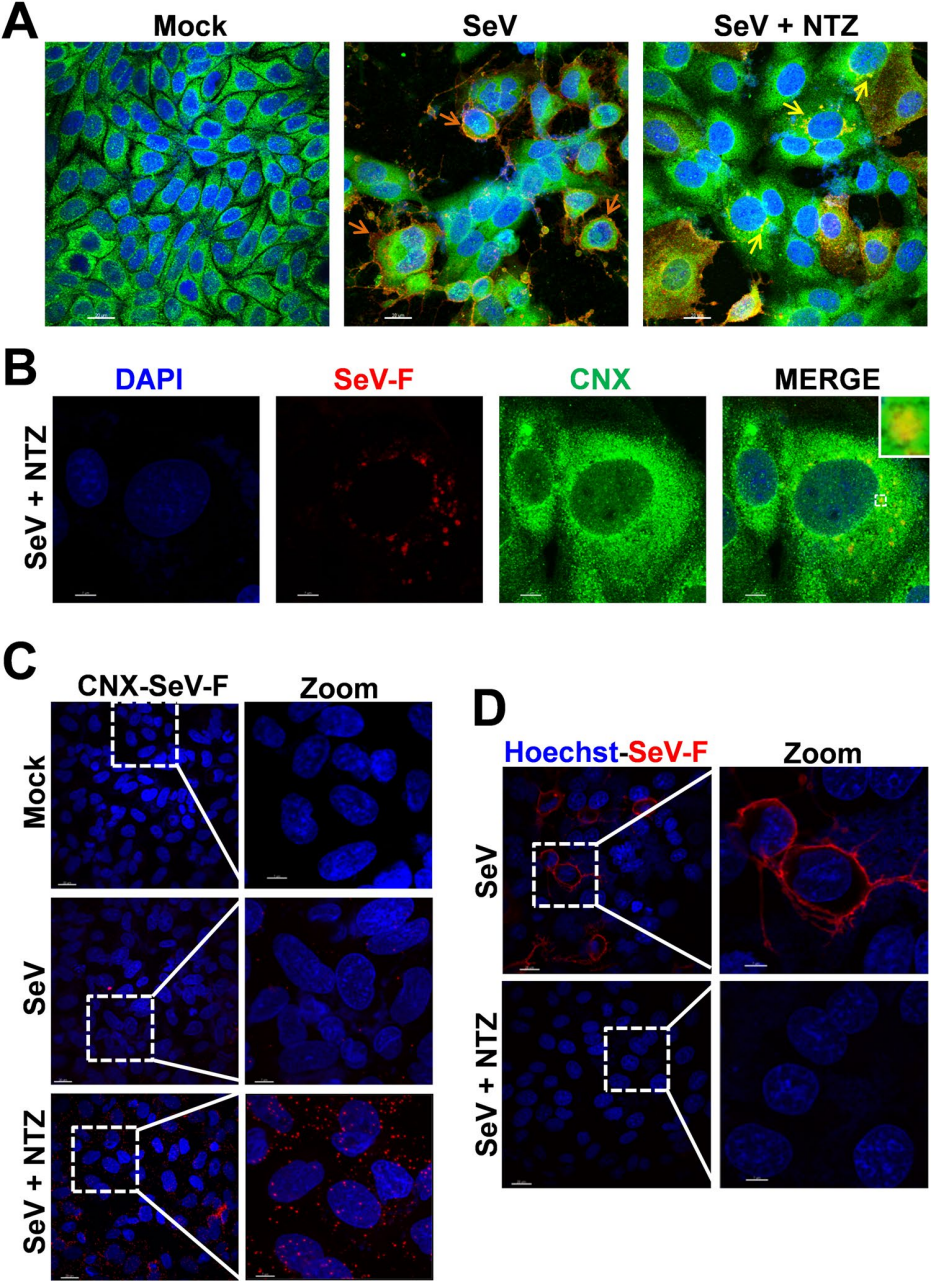
Nitazoxanide alters SeV-F protein intracellular distribution and prevents transport to the host cell surface. (**A**) Confocal images of SeV-F (red) and ER-marker calnexin (CNX) (green) in mock- and SeV-infected AGMK cells treated with NTZ (10 μg/ml) or vehicle for 24 h. Nuclei are stained with DAPI (blue). Merge images are shown. Scale bar, 20 μm. Orange arrows indicate F-protein plasma membrane localization in untreated cells (SeV); yellow arrows indicate perinuclear F-protein aggregates in NTZ-treated cells (SeV + NTZ). **(B)** Confocal images of large SeV-F-protein (red) perinuclear aggregates in SeV-infected AGMK cells treated with NTZ (10 μg/ml) for 24 h. ER-marker calnexin (CNX) (green) is shown. Nuclei are stained with DAPI (blue). Merge images are shown. Scale bar, 7 μm. The enlarged area in the inset highlights F-protein/calnexin colocalization. (**C**) SeV-F/calnexin (CNX) interactions (visualized as red spots) detected at 24 h p.i. by *in situ* proximity ligation assay (PLA) in SeV-infected AGMK cells treated with NTZ (5 μg/ml) or vehicle. Nuclei are stained with DAPI (blue). Scale bar, 20 μm (zoom, 7 μm). (**D**) Confocal images of SeV-F (red) distribution on plasma membrane in SeV-infected AGMK cells treated as in (**C**) and processed for plasma membrane staining, as described in Methods. F-protein could not be detected on the plasma membrane of NTZ-treated cells, despite the presence of high intracellular levels (see Fig. 3C). Scale bar, 20 μm (zoom, 7 μm).

Because of the “stop-start” transcriptional mechanism regulating SeV-mRNA expression^1^, it is unlikely that F-protein depletion could be due to selective inhibition of F-mRNA transcription/translation; we therefore postulated that, as previously described for influenza virus hemagglutinin^10^, NTZ treatment could cause a post-translational modification of F-protein leading to its prompt degradation via ERAD (ER-associated degradation), a sophisticated process for ER-to-cytosol dislocation and ubiquitin/proteasome-mediated degradation of target-proteins^7,15^. However, F-protein reduction in NTZ-treated cells was not prevented by proteasome-inhibition following treatment with the dipeptidyl boronic acid proteasome inhibitor bortezomib (PS-341)^16^ in both AGMK (Fig. 2D) and human A549 (Supplementary Fig. 2C) cells. Surprisingly, the synthesized F-protein, though to a lesser extent, was instead found in the insoluble fraction of NTZ-treated cells (Fig. 3A), suggesting that an alteration in protein maturation could lead to the formation of insoluble F-protein aggregates. Immunomicroscopy analysis in fact showed the presence of large F-protein aggregates in NTZ-treated cells (Figs 3C and 4A,B). F-protein aggregates were found to be mainly localized in the endoplasmic reticulum of NTZ-treated cells, as shown by confocal immunomicroscopy analysis (Fig. 4A,B) and *in situ* proximity ligation assay (PLA) (Fig. 4C), using the ER-resident molecular chaperone calnexin as the ER marker. These aggregates appear to be partially resistant to solubilization under the conditions described, since F-protein levels in the insoluble fraction of NTZ-treated cells were not increased as compared to control (Fig. 3A). Similar results were obtained in the presence of proteasome (lactacystin or MG132) or autophagic-lysosomal system (chloroquine, concanamycin A) inhibitors (data not shown).

Analysis of F-protein plasma membrane levels revealed that SeV-F could not be detected on the host-cell surface in infected NTZ-treated cells (Fig. 4D, Supplementary Fig. 3A) up to 24 hours after infection, whereas large amounts of F-protein were detected in the untreated control cells. Interestingly, similar results were obtained in HeLa cells transiently expressing a FLAG-tagged fusion-protein from respiratory syncytial virus (Fig. 3B and Supplementary Fig. 3B) in the absence of viral infection, suggesting a host-mediated effect of NTZ on fusion proteins of different *Paramyxoviridae* family members.

Correct F-protein folding is a complex dynamic process. Following N-glycosylation in the ER, SeV-F interacts with lectin chaperones calnexin and, to a lesser extent, calreticulin, and with their associated co-chaperone ERp57^5,6^. SeV-F contains 10 highly conserved cysteine-residues at specific positions playing important roles in F-protein folding and function by forming intra- and inter-chain disulfide-bridges^17^, a process largely dependent on ERp57^5,6^. Disulfide-bond formation starts early, and during the folding process native as well as improperly oriented disulfide-bonds may form. The unscrambling of wrong cysteine-bridges into native ones is also assisted by ERp57^18–20^.

ERp57/F-protein and ERp57/calnexin interactions were then investigated by PLA in SeV-infected AGMK and A549 cells. Interestingly, a stronger stable interaction of ERp57 with F-protein, as well as with calnexin, was detected in NTZ-treated as compared to control cells (Supplementary Figs 4A,B and 5A,B). Since it was established that SeV-F interacts with ERp57 transiently under normal conditions, while the interaction is reinforced and prolonged for F-proteins having trouble forming correctly oriented intramolecular disulfide-bonds^5,6^, these observations prompted us to investigate whether thiazolides could interfere with ERp57 activity.

**Figure 5:**
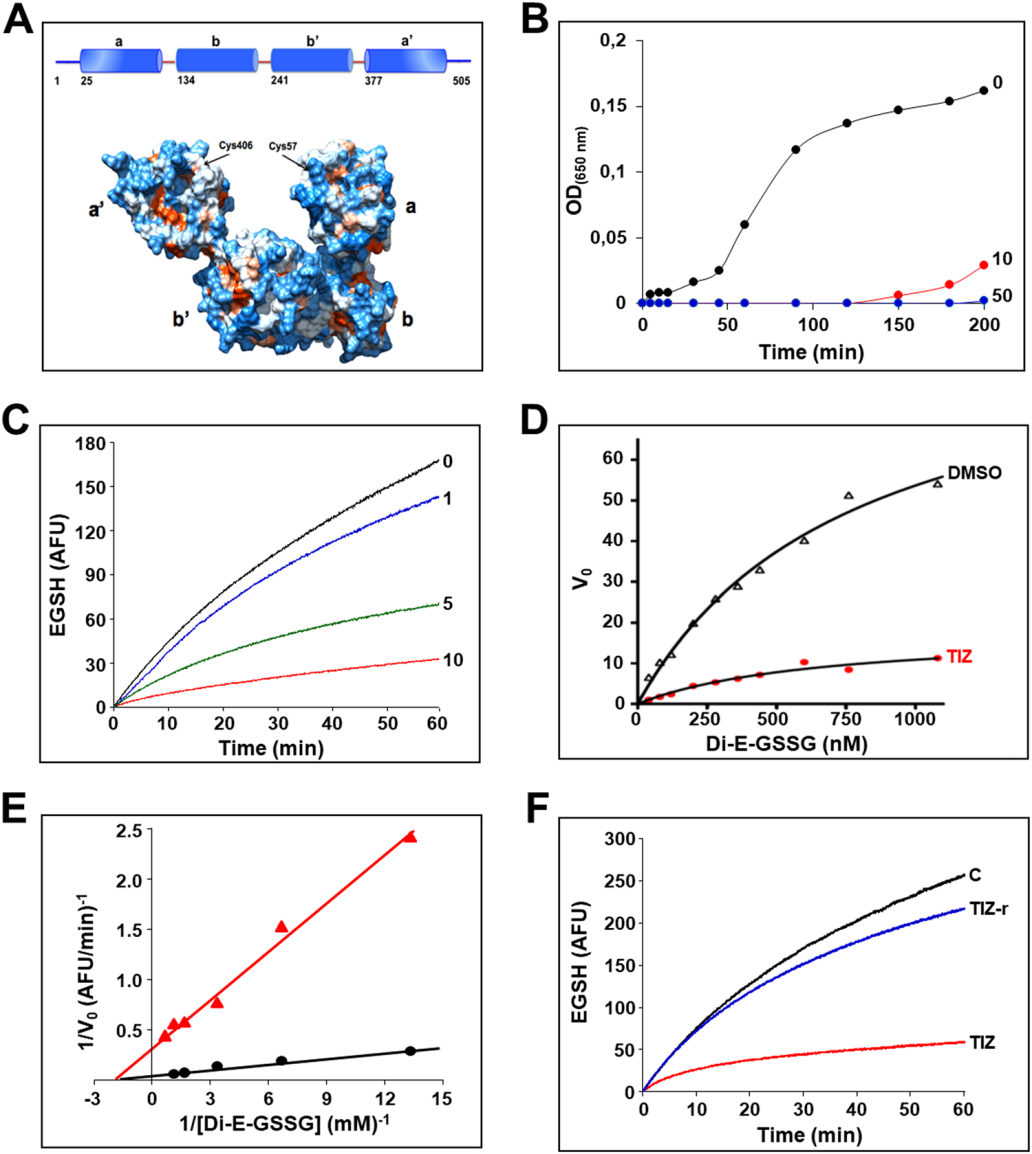
Tizoxanide inhibits ERp57 activity. (**A**) Domain organization (*top*) and surface representation (*bottom*) of ERp57. (**B**) Effect of tizoxanide (10 and 50 μg/ml) on ERp57 disulfide reductase activity, determined by the insulin reduction turbidometric assay, as described in Methods. Curves show the increase in absorbance values (OD) after addition of DTT. For comparison is shown the corresponding control experiment with vehicle only (0, black line). (**C**) Kinetics of dieosin glutathione disulfide (Di-E-GSSG) reduction into fluorescent EGSH catalyzed by ERp57 (20 nM) in the presence of different concentrations (1, 5 and 10 μg/ml) of TIZ or vehicle (0). Data are expressed as arbitrary fluorescence units (AFU). (**D**) The effect of TIZ (10 μg/ml) was evaluated in the di-eosin-GSSG assay using increasing concentrations of substrate (Di-E-GSSG). Michaelis–Menten kinetics were evaluated using GraphPad Prism 5.0 and compared with vehicle only (DMSO). (**E**) Lineweaver-Burk plot of the inhibition of ERp57 disulfide-reductase activity by TIZ (10 μg/ml), at increasing concentrations of Di-E-GSSG. (**F**) Reversibility of ERp57 inhibition by TIZ. ERp57 (400 nM) was incubated with 10 μg/ml TIZ for 15 min, and subsequently diluted 20 times into reaction buffer (TIZ-r). Di-E-GSSG reduction was compared with samples containing 20 nM ERp57 in the presence of 10 μg/ml TIZ (TIZ) or vehicle (**C**). The TIZ-r curve (blue) shows the rapid recovery of enzymatic activity after dilution, as compared to an undiluted sample (red).

ERp57, also known as GRP58 (glucose-regulated protein-58) is a member of the protein disulfide-isomerase (PDI) family coded by the PDIA3 gene, mostly, but not exclusively, localized in the ER^7,18–22^. Like other PDIs, ERp57 is characterized by the presence of two catalytically active thioredoxin-like domains (TLD) containing a cys-gly-his-cys sequence, termed a and a′, which provide ERp57 with its redox properties, and two inactive TLD, termed b and b′ (Fig. 5A, top). ERp57 is a multifunctional protein acting both as oxidoreductase and isomerase, helping ER-glycoproteins to obtain native disulfides by rearranging nonnative linkages. Crystallography studies demonstrate that, like PDI, the four TLD form a twisted “U”-shape structure (Fig. 5A, bottom)^23,24^. The catalytically-active domains are located at the top of the U at the C- and N-termini, while the two noncatalytic domains are localized to the inside surface providing the binding sites for calnexin/calreticulin proline-rich P-domain; in particular, ERp57-b′ needs to associate with calreticulin/calnexin that position lectin-bound misfolded glycoproteins allowing ERp57-mediated catalysis^19,23,24^.

As nitazoxanide is a prodrug and liberates in the blood stream the de-acetyl corresponding derivative tizoxanide, we first investigated the effect of the bioactive NTZ-metabolite TIZ on ERp57 disulfide-reductase activity using the insulin reduction turbidometric assay, as well as a fluorescence-based di-eosin-glutathione disulfide reductase *in-vitro* assay, using purified full-length recombinant human ERp57. TIZ inhibited ERp57-activity in a dose-dependent manner; a potent inhibitory activity was obtained at 10 μg/ml (Fig. 5B–E), a concentration achieved in plasma of nitazoxanide-treated patients^11^. The mode of inhibition was analyzed using the classic Michaelis-Menten formalism (Fig. 5D). Lineweaver-Burk plots (Fig. 5E) showed that TIZ behaves as a non-competitive inhibitor, indicating that the drug does not bind to ERp57 catalytic a/a′-sites, but to a secondary-site; the constant of inhibition K_I_ was calculated to be 1.5 µg/ml. Interestingly, TIZ removal from the reaction mixture readily restored ERp57-activity (Fig. 5F), indicating that TIZ is a reversible-inhibitor of human ERp57. Accordingly, thiazolide removal at 6 h p.i. resulted in reversal of the antiviral activity and attenuation of F-protein insolubilization in SeV-infected cells (Supplementary Fig. 6). Notably, the liver metabolite TIZ-glucuronide, that does not inhibit virus replication, did not affect ERp57 activity (Supplementary Fig. 7). Interestingly, NTZ was reported to also inhibit protozoan *Neospora caninum* PDI activity^25^.

**Figure 6:**
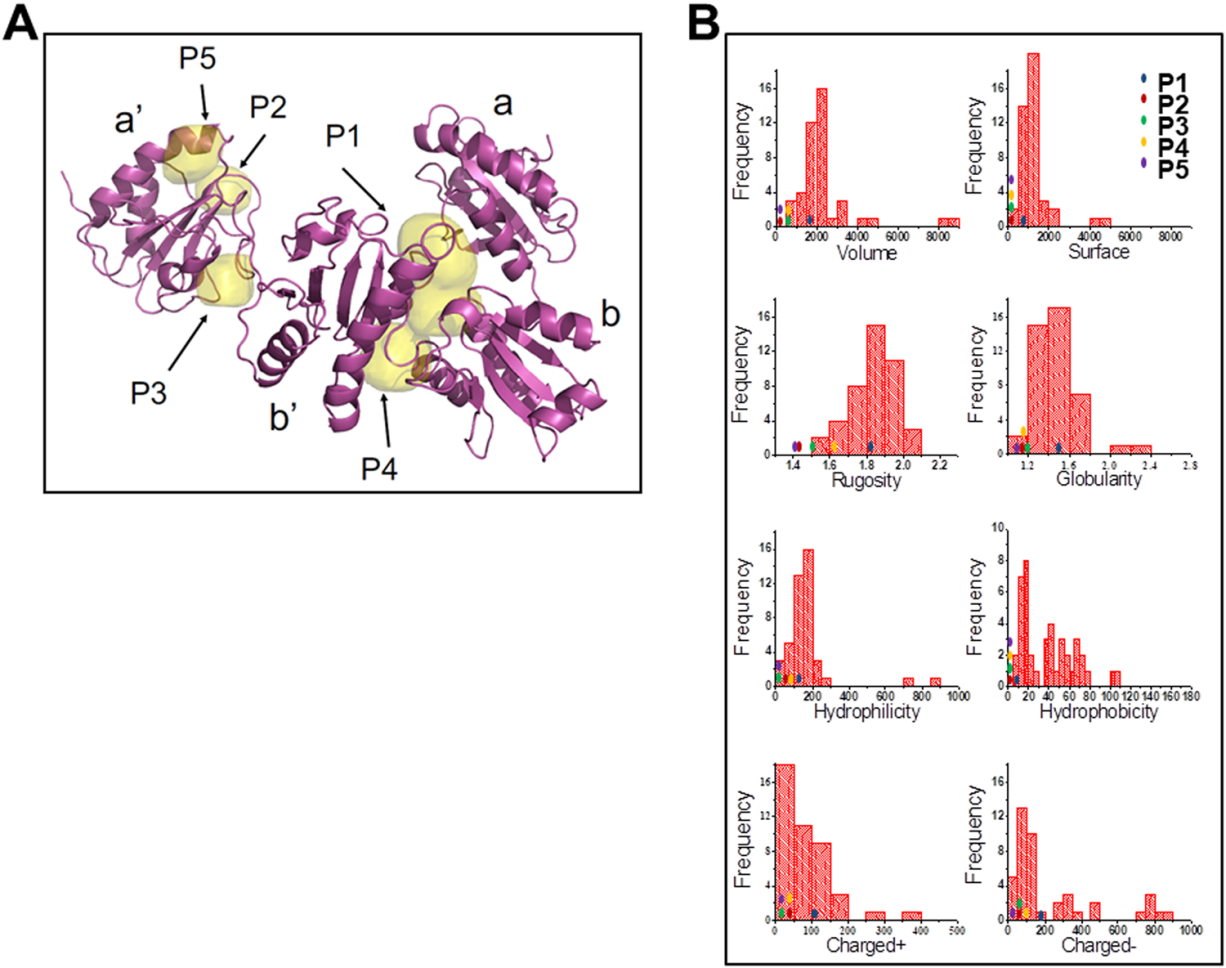
Identification of ERp57 ligand-binding cavities. (**A**) Cavities P1-P5 were detected by FLAPsite on the X-ray structure of the ERp57 complex available from the Protein Data Bank with PDB code 3F8U. ERp57 a, a’, b and b’ domains are indicated. (**B**) ERp57 cavities Volsurf interaction descriptors. Distributions of the Volsurf interaction descriptors for the cavities of the druggable reference set by Cheng *et al*.^27^, and position of P1, P2, P3, P4 and P5 cavities found in ERp57 (colored dots).

Next, ERp57 ligand-binding cavities of ERp57 X-ray structure were mapped by *FLAPsite*^26^. Five possible pockets (P1-P5) were detected (Fig. 6A); among those, the P1-cavity placed at the b/b′-interface^25^ was identified as the putative ERp57-ligand binding-site based on the match of its chemical-physical properties compared to the molecular profile returned by a collection of reference druggable sites^27^. As depicted in Fig. 6B, the P1-cavity, represented by a blue dot, intercepts the most populated ranges of key physico-chemical properties of well investigated druggable binding sites. This knowledge-based approach indicates that the P1-cavity is expected to represent the preferred ERp57 binding site as compared to the other pockets detected.

**Figure 7:**
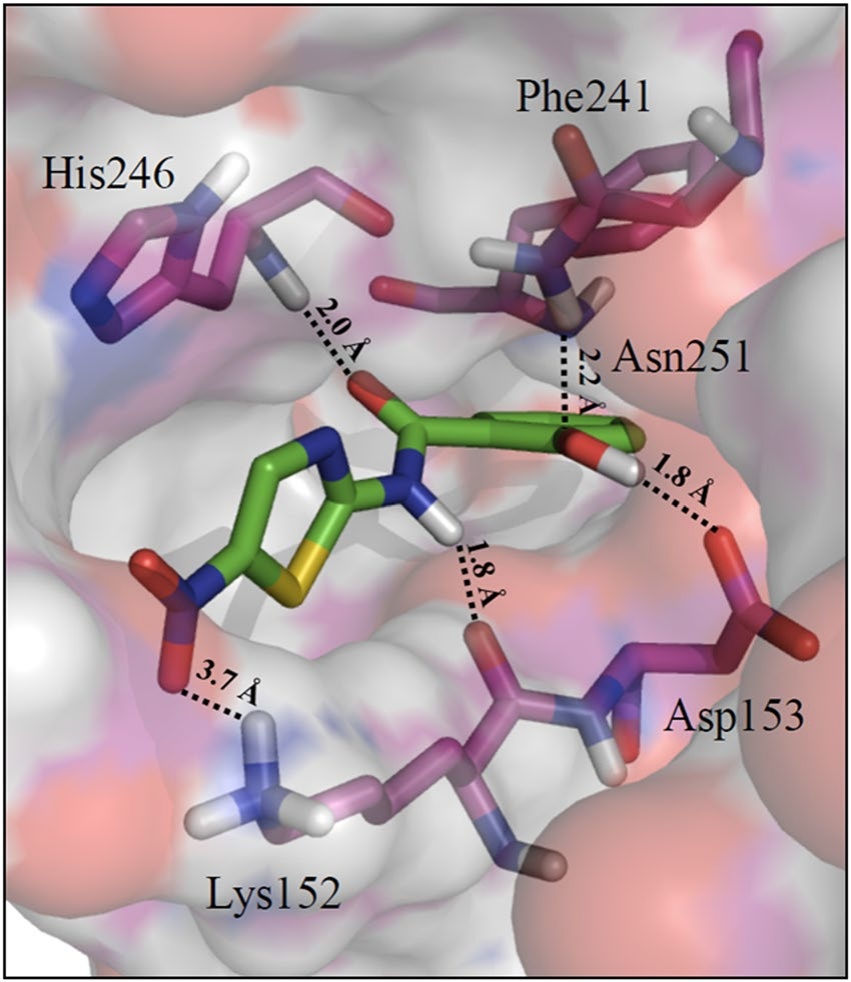
Interaction of tizoxanide with ERp57 P1 cavity. Top-scored pose (−6.397 kcal/mol) of TIZ and key residues of ERp57 P1 pocket are shown in stick, while protein is rendered in cartoon. H-bond interactions are depicted by a dotted line. Only polar hydrogen atoms are shown for clarity.

To gain insight into the molecular interactions behind the remarkable affinity of TIZ towards ERp57, induced-fit docking experiments were carried out within the P1-cavity. The results indicate that TIZ-binding is likely due to the occurrence of a network of well-oriented H-bonds. More specifically, a key residue is Asp153 engaging a charge reinforced H-bond interaction with TIZ phenolic group, whose posing is further ensured by the additional H-bond of Asn251 side-chain (Fig. 7). Other important H-bonds are established by Lys152, which approaches the nitro group of the thiazole ring and the amido linker of TIZ through its charged side-chain and its backbone, respectively. Notably, the inactive NTZ metabolite TIZ-glucuronide (Supplementary Fig. 7) molecular docking was unsuccessful to reproduce the TIZ-binding pose into the P1-cavity.

**Figure 8:**
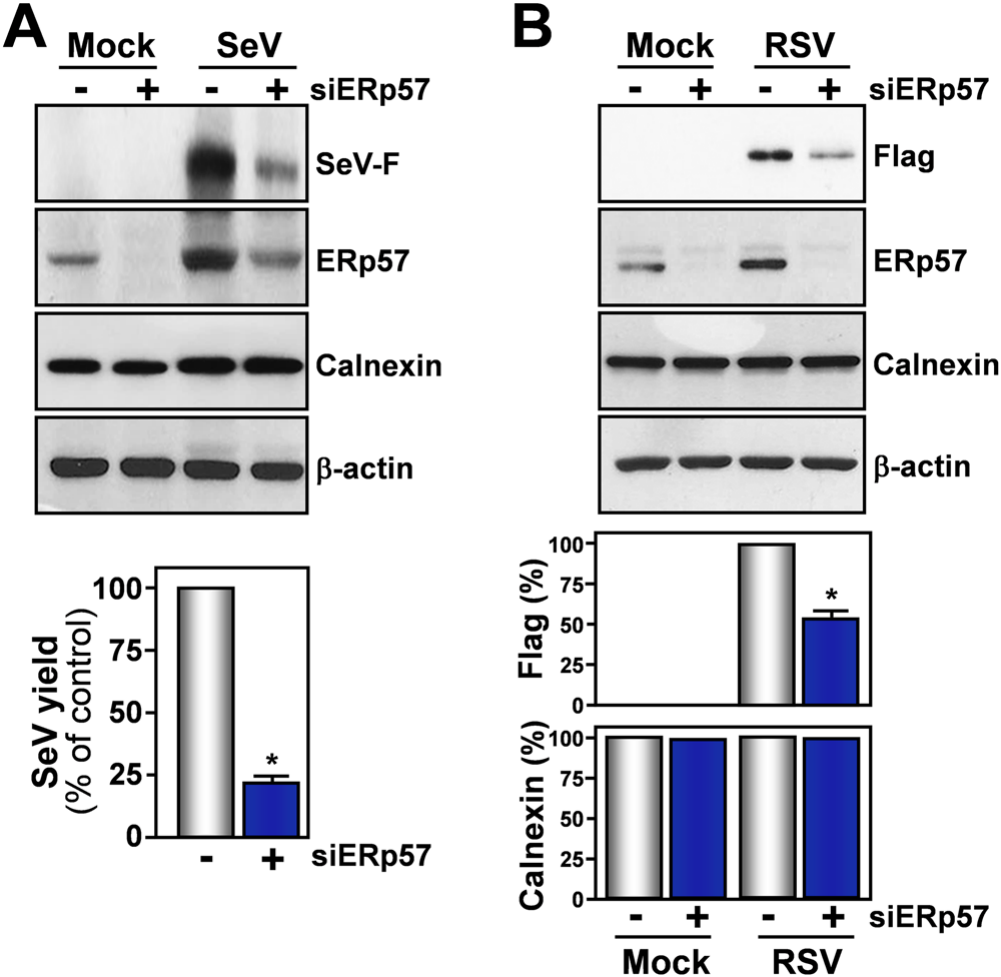
Effect of ERp57 silencing on F-protein levels and SeV replication. **(A)** IB for SeV-F, ERp57, calnexin and β-actin in WCEs from AGMK cells transfected (48 h) with scramble-RNA (−) or ERp57-siRNA (siERp57) (+), and mock-infected or infected with SeV (3 PFU/cell) for 24 h (*top panels*). In parallel samples, virus yield was determined at 24 h p.i. by hemagglutinin titration (*bottom panel*). Data are expressed as percentage of untreated control of quadruplicate samples. Error bars indicate ± S.D. *P < 0.05. (**B**) IB for Flag-RSV-F, ERp57, calnexin and β-actin in WCEs from A549 cells transfected with scramble-RNA (−) or ERp57-siRNA (siERp57) (+), and, after 24 h, transfected with RSV-F/ORF/C-Flag construct (RSV) or pcDNA3 empty vector (Mock) in the presence of si-ERp57 or scRNA (*top panels*). RSV-F (Flag) and calnexin protein levels were quantified as described in Methods, normalized to β-actin levels in the same samples, and expressed as percentage of the relative control. Data represent the mean ± S.D. of three independent experiments (lower panels). *P < 0.05. Full-length blots/gels are presented in Supplementary Fig. 14.

These results suggest that thiazolide-mediated inhibition of ERp57 activity may result in preventing the correct F-protein disulfide-bond architecture leading to F-protein misfolding and aggregation. In order to investigate this possibility, we evaluated the effect of ERp57 down-regulation on SeV infection. ERp57-silencing resulted in a reduction of soluble F-protein levels and, interestingly, caused a significant decrease in virus yield in AGMK cells (Fig. 8A), confirming a critical role of this foldase in SeV-replication. In addition, lower concentrations of NTZ were needed to alter F-protein maturation in ERp57 knock-down cells (Supplementary Fig. 8).

ERp57-silencing also resulted in a significant reduction of F-protein levels in A549 cells transiently expressing FLAG-tagged RSV F-protein (Fig. 8B) in the absence of viral infection.

Altogether these results suggest that, by inhibiting the host foldase, NTZ provokes F-protein misfolding causing the formation of insoluble aggregates, thus impairing F-trafficking to the host plasma membrane, a critical event for biogenesis of mature progeny viruses, as well as for infected cell and adjacent cells fusion. However, we cannot exclude the existence of additional ERp57- dependent or -independent mechanisms that may contribute to the antiviral activity of thiazolides. In the first case, ERp57 inhibition may result in a transient alteration of ER homeostasis. The effect of NTZ on a panel of ER proteins including, in addition to ERp57, ERp72, XBP1, calreticulin and the ER-stress marker GRP78 (glucose-regulated protein-78), was then analyzed in uninfected and SeV-infected AGMK and A549 cells at different times after treatment, and compared to the effect of the ER-stress inducer tunicamycin. Although only a modest increase in the levels of GRP78 was detected in AGMK cells as compared to tunicamycin (Supplementary Fig. 9), it cannot be excluded that an alteration of the ER folding machinery may also affect SeV HN-glycoprotein maturation. The possibility that defects in HN-protein maturation may also participate in thiazolide antiviral activity in SeV-infected cells is currently under investigation. In addition, it should be noted that nitazoxanide was found to inhibit oxidative phosphorylation leading to decreased ATP levels in cancer cells^28,29^. Since metabolic energy is known to be required for correct disulfide bond formation and folding of several proteins in the ER^30–32^, it is possible that lower ATP levels in NTZ-treated cells may contribute to F-protein misfolding in our model. Finally, the fact that nitazoxanide, at clinically relevant concentrations, affected both *Paramyxovirinae* (SeV) and *Pneumovirinae* (RSV) fusion proteins suggests a broad-spectrum activity of the drug.

Among different *Paramyxoviridae* family members, mumps and measles are controlled by an effective vaccination program, whereas there is no effective cure or prevention for most paramyxoviruses. In addition, the large number of novel paramyxoviruses recently identified in bats and rodents^33^, as well as the increasing number of lethal henipavirus infections and human-to-human transmission^2^, highlight the need for novel therapeutic strategies against these pathogens. Because of its critical role in cell entry, the fusion-protein represents an attractive target for novel antivirals^34^. Most of the current attempts to block F-protein function are aimed at halting the cascade of conformational changes driving membrane fusion through stabilization of the pre-fusion conformation of virion F-trimers by designing specific peptide inhibitors, or developing selective or cross-reactive neutralizing antibodies^35–37^. We now propose a novel approach to target paramyxovirus fusion proteins by drug-directed misfolding of newly synthesized F-protein.

Nitazoxanide has been used for decades in the clinic as a safe and effective antiprotozoal/antimicrobial drug; more recently, its antiviral activity was shown in a large number of adults and children infected with hepatitis-C virus, rotavirus and A/B influenza virus^10–14^. Our results now suggest that nitazoxanide may be effective also against paramyxovirus infection. In addition, since several viral glycoproteins, including influenza virus hemagglutinin^38^, are known to depend on ERp57 for correct disulfide-bond architecture, inhibition of ERp57 may play a more general role in the broad-spectrum antiviral activity of nitazoxanide.

## Methods

### Cell Culture, Treatment and Transfections

African green monkey (*Cercopithecus aethyops sabeus*) kidney (AGMK) 37RC cells (a kind gift from Arrigo Benedetto, Centre of Virology, OO.RR. San Camillo-Forlanini, Rome, Italy)^39^ and human A549 alveolar type II-like epithelial cells and cervical carcinoma HeLa cells (American Type Culture Collection) were grown at 37°C in a 5% CO_2_ atmosphere in RPMI-1640 (AGMK, A549) or DMEM (HeLa) medium (EuroClone), supplemented with 10% fetal calf serum (FCS), 2 mM glutamine and antibiotics. Nitazoxanide [2-acetyloxy-*N*-(5-nitro-2-thiazolyl)benzamide, Alinia], tizoxanide, tizoxanide-glucuronide (TIZ-Glc) and second-generation thiazolides RM4820, RM4832, RM4848, RM5038 (Romark Laboratories, L.C.), proteasome inhibitor bortezomib, glycosylation inhibitor tunicamycin (Sigma-Aldrich), dissolved in DMSO stock solution, were diluted in culture medium, added to infected cells after a one-hour virus adsorption period, and maintained in the medium for the duration of the experiment, unless differently specified. Controls received equal amounts of DMSO vehicle, which did not affect cell viability or virus replication. Cell viability was determined by the 3-(4,5-dimethylthiazol-2-yl)-2,5-diphenyltetrazolium bromide (MTT) to MTT formazan conversion assay (Sigma-Aldrich), as described^40^. The 50% lethal dose (LD_50_) was calculated using Prism 5.0 software (GraphPad Software Inc.). Microscopical examination of mock-infected or virus-infected cells was performed daily to detect virus-induced cytopathic effect and possible morphological changes and/or cytoprotection induced by the drug. Microscopy studies were performed using a Leica DM-IL microscope and images were captured on a Leica DC 300 camera using Leica Image-Manager500 software.

For transfection experiments, semiconfluent monolayers of HeLa or A549 cells were transiently transfected with a pCMV-driven construct containing the gene expressing the F-protein of human respiratory syncytial virus (RSV, subtype A, strain A2) linked to a FLAG-tag [pCMV3-RSV-F(A-A2)C-FLAG (RSV-F/ORF/C-Flag), Sino Biological Inc.] or pcDNA3 empty vector as control. Transfections were performed using jetPRIME Transfection Reagent (Polyplus transfection), according to the manufacturer’s instructions.

### Virus preparation, infection and titration

Preparation of Sendai virus (SeV) by allantoic inoculation of 10-day-old embryonated eggs (Charles River Laboratories Italia S.r.l) has been previously described^39^. Protocol for SeV production was performed in accordance with Italian dLgs 116/92. After 72 hours at 37 °C, the allantoic fluid was harvested, clarified by centrifugation at 4,000 × g for 10 minutes, aliquoted and stored at −80 °C. Virus titers were determined by plaque assay^41^. For virus infection, confluent AGMK and A549 cell-monolayers were infected with SeV for 1 hour at 37 °C at a multiplicity of infection (MOI) of 3 PFU (Plaque Forming Unit)/cell, unless differently specified. After the adsorption period, the viral inoculum was removed, and cell-monolayers were washed three times with phosphate-buffered saline (PBS). Cells were maintained at 37 °C in RPMI-1640 medium containing 2% FCS. For multistep virus growth curves, confluent AGMK/A549 cell monolayers were infected with SeV for 1 h at 37 °C at an MOI of 0.01 PFU/cell. After the 1 h adsorption period, the viral inoculum was removed and, after repeated washing with PBS, cells were maintained at 37 °C in RPMI-1640 culture medium containing 0.1% bovine serum albumin (BSA) and L-1-tosylamide-2-phenylethyl chloromethyl ketone (TPCK)-treated trypsin (1 μg/ml) (Sigma-Aldrich). Virus yield was determined 24 and 48 h p.i. by hemagglutinin (HA) titration or by plaque assay, as described^40,41^. The IC_50_ (50% inhibitory concentration) of the compounds tested was calculated using Prism 5.0 software.

### Metabolic Labeling, Analysis of Protein Synthesis and Western Blot

Cells were labeled with 10 μCi/ml of [^35^S]-methionine-cysteine ([^35^S]-Met/Cys, Easy-Tag™ Express Protein-labeling mix, PerkinElmer) for the indicated times after 30 min starvation in methionine/cysteine-free medium. [^35^S]-Met/Cys incorporation was determined after cell lysis in Laemmli sample buffer^42^. Samples containing the same amount of radioactivity or the same amount of total lysate were separated by SDS/PAGE (3% stacking gel, 10% resolving gel) and processed for autoradiography, as described^42^. Autoradiographic patterns were visualized and quantified in Typhoon-8600 Imager (Molecular Dynamics Phosphor-Imager^TM^), and images were acquired using ImageQuant software (Amersham Pharmacia Biotech)^43^.

For analysis of soluble/insoluble proteins whole-cell extracts (WCE) were prepared after lysis in High Salt Buffer (HSB) (50 mM Tris-HCl pH 7.5, 350 mM NaCl, 1 mM MgCl_2_, 0.5 mM EDTA, 0.1 mM EGTA, 1% NP-40 and 20% glycerol) supplemented with 20 mM β-glycerolphosphate, 1 mM p-NPP (p-nitrophenyl phosphate), 1 mM Na_3_VO_4_, 1 mM PMSF and protease-inhibitors cocktail (PIC; Roche Applied Science). Briefly, cells were washed twice with ice-cold PBS and then lysed in HSB (80 μl). After one cycle of freeze and thaw, and centrifugation at 16,000 × g (10 min at 4 °C), supernatant (soluble) and pellet (insoluble) fractions were collected. Insoluble fractions were solubilized in 60 μl of Buffer-S (50 mM Tris-HCl pH 8.5, 1% SDS and protease inhibitors) by sonication on ice, using an ultrasonic UP50H processor (Hielscher) (40% amplitude, pulse mode: 6 × 10 sec, 15 sec pauses)^44^.

For Western blot analysis, cell extracts (20 μg) were separated by SDS-PAGE and blotted to nitrocellulose as described^44^, and filters were incubated with the following antibodies: monoclonal anti-SeV-F (αF-γ236; ID Pharma), anti-ERp57 (MaP.ERp57, Santa Cruz), anti-ubiquitin (P4D1, Santa Cruz), anti-α-tubulin (B-5-1-2, Sigma-Aldrich), anti-KDEL (Enzo Life Sciences) antibodies; polyclonal anti-ERp57 (Merck-Millipore), anti-FLAG (DYKDDDDK Tag, Cell Signaling Technology, Inc.), anti-histone H3 (Abcam), anti-calnexin (StressGen), anti-β-actin (Sigma-Aldrich), anti-calreticulin (StressGen), anti-XBP1 and anti-ERp72 (Enzo Life Sciences) antibodies, followed by decoration with peroxidase-labeled anti-rabbit IgG or anti-mouse IgG (SuperSignal detection kit; Pierce). Quantitative evaluation of proteins was determined by Versadoc 1000 (Bio-Rad) analysis using the Quantity One software program (Bio-Rad Laboratories).

### siRNA interference

siRNA duplex sequences, si-ERp57 (5′-TCCAGCCAACAAGAAGCTAAA-3′), and the scrambled control (scRNA, 5′-GACAACAGATCACCGATACAA-3′) sequence were purchased from QIAGEN. Transfections were performed using jetPRIME Transfection Reagent (Polyplus-transfection), according to the manufacturer’s instructions. Briefly, for ERp57 silencing, cells were plated on 35-mm wells (1.5 × 10^5^ cells/well) and, after 18 h, were transfected with 50 nM of the indicated siRNA or scrambled control. After 24 h cells were washed twice with culture medium and transfections were repeated as above. For SeV infection experiments, at 48 h after transfection siRNAs and scRNA were removed, and cells were washed twice with culture medium and subjected to SeV infection (3 PFU/cell), as described above. For RSV-F experiments, at 24 h after transfection siRNAs and scRNA were removed, cells were washed twice with culture medium and subjected to RSV-F/ORF/C-Flag or pcDNA3 transfection for 24 h in the presence of si-ERp57 or scRNA.

### Immunofluorescence microscopy

SeV-infected AGMK or A549 cells, and RSV-F-transfected HeLa cells grown on coverslips were fixed with 2% or 4% paraformaldehyde in PBS for 20 minutes at room temperature at 16 h p.i. Mock-infected or mock-transfected cells were processed similarly. Cells were incubated with anti-SeV-F monoclonal or anti-FLAG polyclonal antibodies for 1 h at 37 °C for plasma membrane staining, or were permeabilized with 0.1% TritonX-100-PBS for 10 min at room temperature and then incubated with monoclonal anti-SeV-F, or polyclonal anti-calnexin, or anti-FLAG antibodies for 1 h at 37 °C, followed by decoration with Alexa Fluor 488- or 555-conjugated antibodies (Molecular Probes-Invitrogen). Control incubations demonstrated non-cross-reactivity between the anti-immunoglobulin conjugates, or between the anti-immunoglobulin conjugate and the irrelevant primary antibody. Nuclei were stained with 4′,6-diamidino-2-phenylindole (DAPI) or Hoechst 33342 (Molecular Probes, Invitrogen). Images were captured using an Olympus FluoView FV-1000 confocal laser scanning system (Olympus America Inc.) based on Olympus IX81 inverted microscope equipped with Olympus Plan-Apochromat 60× oil-immersion objective. Images (800 × 800 pixels resolution) were analyzed using Imaris 6.2 software (Bitplane). Images shown in all figures are representative of at least five random fields (scale-bars are indicated).

### Proximity ligation assay (PLA)

For PLA-assay, cells were grown on coverslips and processed as described above. After incubation with the primary antibodies, Duolink *in situ* PLA (Sigma-Aldrich) was performed as described^44^. Briefly, PLA probes were incubated for 1 h at 37 °C, followed by hybridization, ligation (30 min at 37 °C) and amplification (100 min at 37 °C). Nuclei were stained with DAPI in Duolink *In Situ* Mounting Medium (Sigma-Aldrich). Antibodies used are: monoclonal anti-ERp57 and anti-SeV-F, polyclonal anti-ERp57, anti-FLAG and anti-calnexin. Images were captured using an Olympus Fluoview FV1000 confocal laser scanning system (Olympus America Inc.) described above. Images (800 × 800 pixels resolution) were analyzed using Imaris 6.2 software (Bitplane). Images shown in all figures are representative of at least five random fields (scale-bars are indicated).

### Disulfide reductase (DR) activity assay

ERp57 DR activity was determined by the insulin reduction turbidometric assay, as well as by a fluorescence-based dieosin glutathione disulfide (Di-E-GSSG) reductase *in-vitro* assay. For the insulin reduction assay, ERp57-catalyzed reduction of insulin was assayed by measuring the aggregation of reduced insulin chains in the presence of DTT, as detected at an optical density (OD) at λ = 650 nm^45,46^; the reaction mixture contained 100 mM potassium phosphate (pH 7.0), 2 mM EDTA, 200 nM of full-length recombinant human ERp57 (rhERp57, Abcam), 1 mg/ml (170 μM) of insulin (Sigma), 500 μM DTT in a total volume of 100 μl in a 96-well plate. For TIZ activity testing, rhERp57 was preincubated for 15 min at room temperature with different concentrations of TIZ. The increase in insulin aggregation was monitored at 25 °C for 3 h. ERp57 activity was determined by the following formula: OD_DMSO_ = (OD[DMSO + rhERP57 + insulin + DTT] − OD[DMSO + insulin + DTT]); OD_TIZ_ = (OD[TIZ + rhERP57 + insulin + DTT] − OD[TIZ + insulin + DTT])^45^.

ERp57 DR activity was also determined by sensitive fluorescence assay using Di-E-GSSG (Cayman Chemical) as a fluorogenic probe^47^. DR activity was assayed in Reaction Buffer (RB: 0.1 M potassium phosphate, 2 mM EDTA, pH 7.0), by adding 20 nM rhERp57 to 150 nM of Di-E-GSSG, unless differently specified, in the presence of 5 μM DTT. ERp57 reductase activity cleaves the S-S bond of Di-E-GSSG resulting in the release of fluorescent 2-eosin-GSH (EGSH). For thiazolide activity testing, rhERp57 was pre-incubated for 15 min at room temperature with different concentrations of TIZ, TIZ-Glc or DMSO vehicle in RB, unless differently specified. The increase in fluorescence was monitored at λ = 545 nm with excitation at λ = 525, at 25 °C with continuous stirring, and expressed as arbitrary fluorescence units (AFU). The degree of inhibition was determined by the relative amount of fluorescent EGSH formed over a fixed time period, or by the initial velocity of the reaction (AFU formed per min). Michaelis-Menten analysis was made using 20 nM rhERp57 in the presence of TIZ (10 μg/ml) or vehicle as described above. Ten-point response curves were generated using increasing concentrations (40-1250 nM) of substrate (Di-E-GSSG). Enzyme kinetics analysis was performed using GraphPad Prism 5.0.

### Identification of ERp57 cavities

The *FLAPsite* algorithm^27^ was used for identification of cavities in the Tapasin/ERp57 complex (PDB entry: 3F8U)^48^. The procedure starts by embedding the target protein into a grid with a spatial resolution of 1.0 Å. The detection routine through the GRID H probe^49^ identifies pocket points. The next step focuses on those grid points that are located within a distance of 4 Å from the closest protein atom, excluding protein surface points. For the remaining points a buriedness-index is calculated. The buriedness-index is also weighted by hydrophobicity computed using the GRID hydrophobic DRY probe. Points with a buriedness-index lower than a pre-determined threshold are discarded. The remaining points are processed through two morphological operations, namely *erosion* to remove small anomalies (decreasing the size of the cavity) and *dilation* to fill holes and connect areas (increasing the size of the cavity). By using the GRID hydrophobic probe (DRY), hydrophobic cavities that are usually bonded by drugs are prioritized.

### Druggability assessment

The druggability of all the detected cavities was assessed by a knowledge-based approach. In particular, the computed chemical-physical properties were compared with those of well-known druggable active sites. We collected a reference dataset containing 43 target structures defined as ‘druggable’ by Cheng *et al*.^27^. The cavities for each target were detected by the FLAPsite tool. Volsurf descriptors (Molecular Interactions Fields – MIFs)^50^ were used to characterize either the ERp57 cavities or each pocket in the reference druggable target dataset. Volume, surface, rugosity, globularity, hydrophobicity, hydrophilicity and charged residues descriptors were calculated. Pocket volume and surface represent the water-excluded volume and the accessible surface respectively, traced out by the GRID OH2 probe (water probe). Rugosity and globularity represent the presence of wrinkles or creases on the pocket surface and the degree of sphericity of the pocket, respectively. Hydrophobicity and hydrophilicity were quantified using the GRID probes DRY and OH2 respectively. The hydrophobic probe is used to mimic the pocket attractive hydrophobic interactions, while the water probe is used to mimic solvation-desolvation processes.

GRID probes N1+ and O− are instead suitable probes used to detect charge-charge electrostatic interactions but also polar interactions generated by pocket residues. A comparative analysis of the ERp57 cavities against the well-known druggable active sites reference dataset was performed by plotting distributions of the corresponding chemical-physical properties^51,52^.

### Docking studies

Flexible receptor docking studies were performed using a multi-stage induced fit docking protocol (IFD) available from the Schrödinger Suite v2016-4^53^. As a first step, the ligands were subjected to LigPrep to properly generate all the tautomers and ionization states at a pH value equal to 7.0 ± 2.0. A cubic docking grid placed on the centroid of residues defining the detected cavity (i.e. residues 152, 153, 154, 181, 183, 208, 210, 211, 240, 241, 245, 246, 248, 250, 251, 254, 267, 268, 269, 270, 306) was used. Notably, an inner box having a side equal to 10 Å and an automatic outerbox were employed. In the first stage, the van der Waals radii of the protein and the ligand were scaled by a factor of 0.5 and ligands were docked using the default Glide Extra Precision (XP) mode. Next, Prime was used to predict and optimize the selected protein side chains. The poses were scored and filtered, and redocked using the Glide XP mode. All docking simulations were performed using the default Force Field OPLS_2005^54^.

### Statistical analysis

Statistical analysis was performed using Student’s *t* test for unpaired data. The data are expressed as the means ± S.D. of duplicate samples. *P* values of < 0.05 were considered significant. All the results and images shown in this manuscript are representative of at least three independent experiments with similar results.

## Electronic supplementary material

Supplementary Information
