# Supplementary material for: Nitazoxanide inhibits paramyxovirus replication by targeting the Fusion protein folding: role of glycoprotein-specific thiol oxidoreductase ERp57
Source: Sci Rep. 2018 Jul 11;8:10425. doi: 10.1038/s41598-018-28172-9 (PMC6041319; doi:10.1038/s41598-018-28172-9)
Supplement: Supplementary file 1 — Supplementary Information [file 41598_2018_28172_MOESM1_ESM.pdf]

Supplementary Information

Nitazoxanide inhibits paramyxovirus replication by targeting the Fusion protein folding: role of glycoprotein-specific thiol oxidoreductase ERp57

Sara Piacentini, Simone La Frazia, Anna Riccio, Jens Z. Pedersen, Alessandra Topai, Orazio Nicolotti, Jean-Francois Rossignol & M. Gabriella Santoro

A

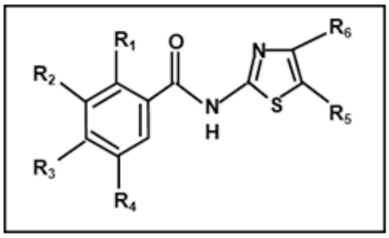

B

Compound	Substituents		IC_{50} [μg/ml]	LD_{50} [μg/ml]
**Nitazoxanide**	R_1=$OCOCH_3$	R_2=H	**0.30 ± 0.01**	**>50**
	R_3=H	R_4=H		
	R_5=NO_2	R_6=H		
**Tizoxanide**	R_1=OH	R_2=H	**0.50 ± 0.01**	**>50**
	R_3=H	R_4=H		
	R_5=NO_2	R_6=H		
**RM4820**	R_1=$OCOCH_3$	R_2=H	**0.40 ± 0.02**	**>50**
	R_3=H	R_4=H		
	R_5=Br	R_6=H		
**RM4832**	R_1=OH	R_2=H	**0.50 ± 0.04**	**>50**
	R_3=H	R_4=H		
	R_5=Br	R_6=H		
**RM4848**	R_1=OH	R_2=H	**0.40 ± 0.03**	**>50**
	R_3=H	R_4=H		
	R_5=Cl	R_6=H		
**RM5038**	R_1=$OCOCH_3$	R_2=H	**0.50 ± 0.02**	**>50**
	R_3=H	R_4=H		
	R_5=Cl	R_6=H		

Supplementary Table 1. Antiviral activity of thiazolides against Sendai virus. A, General structure of thiazolides. **B,** Evaluation of the antiviral activity of the second generation thiazolides RM4820, RM4832, RM4848, and RM5038 as compared to nitazoxanide (NTZ) and tizoxanide (TIZ). AGMK cells mock-infected or infected with SeV under single-step conditions (3 PFU/cell) were treated with different concentrations (0.01, 0.1, 1, 10, and 50 μg/ml) of the compounds immediately after the virus adsorption period. Cell viability and virus yield were determined at 24h p.i. by MTT assay and hemagglutination assay, respectively. Data, expressed as IC_{50} (50% inhibitory concentration) and LD_{50} (50% lethal dose), represent the means±S.D. of triplicate experiments. Lack of SD in the LD_{50} column indicates that values were identical.

Supplementary Figure Legends

Supplementary Figure 1. Antiviral activity of tizoxanide in SeV-infected AGMK cells. A,B, AGMK cells mock-infected or infected with SeV under single-step (3 PFU/cell, *top panels*) and multistep (0.01 PFU/cell, *bottom panels*) conditions were treated with different concentrations of nitazoxanide (NTZ) (**A**), tizoxanide (TIZ) (**B**), or vehicle immediately after the adsorption period. Virus yield (O) was determined at 24h (single-step) or 48h (multistep) p.i. by hemagglutinin (HA) titration. Data, expressed as HAU/ml, represent the means±S.D. of quadruplicate samples. *=*P*<0.05; **=*P*<0.01. Cell viability (24h) of mock-infected cells (Δ) was determined by MTT assay (n=4). Absorbance (O.D.) of converted dye was measured at λ 570nm. **C**, AGMK cells were infected and treated as in (**A,B**). Virus yield was determined in quadruplicate samples at 24h (3 PFU/cell) or 48h (0.01 PFU/cell) p.i. by HA titration. TIZ and NTZ IC_{50} (50% inhibitory concentration) was calculated using Prism 5.0 software. Selectivity Indexes (SI) are shown.

Supplementary Figure 2. Thiazolides inhibit SeV replication in human lung A549 cells. A,B, Human alveolar type II-like epithelial A549 cells mock-infected or infected with SeV under single-step (3 PFU/cell, *top panels*) and multistep (0.01 PFU/cell, *bottom panels*) conditions were treated with different concentrations of nitazoxanide (NTZ) (**A**), tizoxanide (TIZ) (**B**) or vehicle immediately after the adsorption period. Virus yield (O) was determined at 24h (single-step) or 48h (multistep) p.i. by HA titration. Data, expressed as HAU/ml, represent the means±S.D. of quadruplicate samples. *=*P*<0.05; **=*P*<0.01. Cell viability (24h) of mock-infected cells (Δ) was determined by MTT assay (n=4). Absorbance (O.D.) of converted dye was measured at λ 570nm. **C**, Immunoblot analysis for SeV-F, ubiquitin and β-actin in whole-cell extracts of mock-infected and SeV-infected A549 cells treated for 24h with NTZ (5 μg/ml), proteasome inhibitor bortezomib (BTZ) (25 nM), glycosylation inhibitor tunicamycin (TM) (5

μg/ml) or vehicle. A decrease in ubiquitinated protein levels is noted in bortezomib-treated cells in the presence of NTZ. Full-length blots/gels are presented in Supplementary Figure 15.

Supplementary Figure 3. Nitazoxanide inhibits SeV-F and RSV-F glycoprotein translocation to the cell surface. **A,** Levels of plasma membrane SeV-F glycoprotein (*red*) were detected at 24h p.i. by indirect immunofluorescence in SeV-infected A549 cells treated with 5 μg/ml NTZ or vehicle. Nuclei are stained with Hoechst (*blue*). Images were captured using an Olympus Fluoview FV1000 confocal laser scanning system. Merge and zoom images are shown. Scale bar, 20 μm (zoom, 7 μm). **B**, Confocal images of Flag-RSV-F (red) distribution on plasma membrane in HeLa cells transfected with RSV-F/ORF/C-Flag tag construct (RSV-F) or pcDNA3 empty vector (Control), and treated as in (**A**). Nuclei are stained with Hoechst (blue). Merge and zoom images are shown. Scale bar, 20 μm (zoom, 7 μm).

Supplementary Figure 4. Nitazoxanide alters ERp57 interaction with SeV fusion protein. SeV-F/ERp57 interactions (visualized as red spots) detected at 24h p.i. by PLA in SeV-infected AGMK (**A**) and A549 (**B**) cells treated with 5 μg/ml NTZ or vehicle. Nuclei are stained with DAPI (blue). Merge and zoom images are shown. Scale bar, 20 μm (zoom, 7 μm).

Supplementary Figure 5. Nitazoxanide alters ERp57/calnexin interactions in SeV-infected cells. ERp57/calnexin (CNX) interactions (visualized as red spots) detected at 16h p.i. by PLA in SeV-infected AGMK (**A**) and A549 (**B**) cells treated with 5 μg/ml NTZ or vehicle. Nuclei are stained with DAPI (blue). Merge and zoom images are shown. Scale bar, 20 μm (zoom, 7 μm).

Supplementary Figure 6. Reversal of nitazoxanide antiviral activity in SeV-infected cells. **A**, AGMK cells mock-infected or infected with SeV (3 PFU/cell) were treated with NTZ (10 μg/ml) or vehicle after virus adsorption. At 6h p.i. in 50% of the cultures the drug was removed,

and cell monolayers were washed three times with phosphate-buffered saline and incubated in culture medium in the presence of vehicle; NTZ was kept in the remaining 50% of the cultures. At 24h p.i., whole-cell extracts were analyzed for SeV-F and β-actin levels by Western blot. **B**, In parallel samples, virus yield was determined at 24h p.i. by HA titration. Data, expressed as HAU/ml, represent the means±S.D. of quadruplicate samples. *=*P*<0.01. Full-length blots/gels are presented in Supplementary Figure 15.

Supplementary Figure 7. Tizoxanide glucuronide does not affect SeV replication and ERp57 activity. **A**, Structure of tizoxanide glucuronide (TIZ-Glc). **B**, AGMK cells were mock-infected or infected with SeV (3 PFU/cell) and treated with different concentrations of TIZ (*top panel*), TIZ-Glc (*bottom panel*) or vehicle immediately after the adsorption period. Virus yield (O) was determined in quadruplicate samples at 24h p.i. by HA titration. Data, expressed as HAU/ml, represent the means±S.D. of quadruplicate samples. *=*P*<0.05; **=*P*<0.01. Cell viability (24h) of mock-infected cells (Δ) was determined by MTT assay (n=4). Absorbance (O.D.) of converted dye was measured at λ 570nm. **C**, Kinetics of dieosin glutathione disulfide (Di-E-GSSG) reduction into fluorescent EGSH catalyzed by ERp57 (20 nM) in the presence of 30 μM TIZ, 30 μM TIZ-Glc or vehicle (C). Data are expressed as arbitrary fluorescence units (AFU).

Supplementary Figure 8. Effect of ERp57 silencing on F-protein levels in thiazolide-treated SeV-infected cells. IB for SeV-F, ERp57 and β-actin in WCEs from AGMK cells transfected (48h) with scramble-RNA (scRNA) or ERp57-siRNA (siERp57), and mock-infected (M) or infected with SeV (3 PFU/cell) and treated with different concentrations of NTZ, 1 μg/ml tizoxanide (TIZ) or vehicle (-) immediately after the adsorption period for 16h. Full-length blots/gels are presented in Supplementary Figure 16.

Supplementary Figure 9. Effect of nitazoxanide on the level of GRP78 and different ER proteins in SeV-infected cells. IB for GRP78 (anti-KDEL antibodies), ERp57, ERp72, calreticulin, XBP1, SeV-F and α-tubulin in WCEs from AGMK and A549 cells uninfected (**A**), or infected with SeV (3 PFU/cell) or mock-infected (**B**) and treated with 5 μg/ml NTZ, 2.5 μg/ml tunicamycin (TM) or vehicle (C) for different times. In SeV-infected cells treatment was started immediately after the adsorption period and continued for different times post infection (p.i.). Full-length blots/gels are presented in Supplementary Figures 17 to 19.

Supplementary Figures 10-19. Full-length blots/gels of data presented in Figure 2, Figure 3, Figure 8, and in Supplementary Figures 2, 6, 8 and 9.

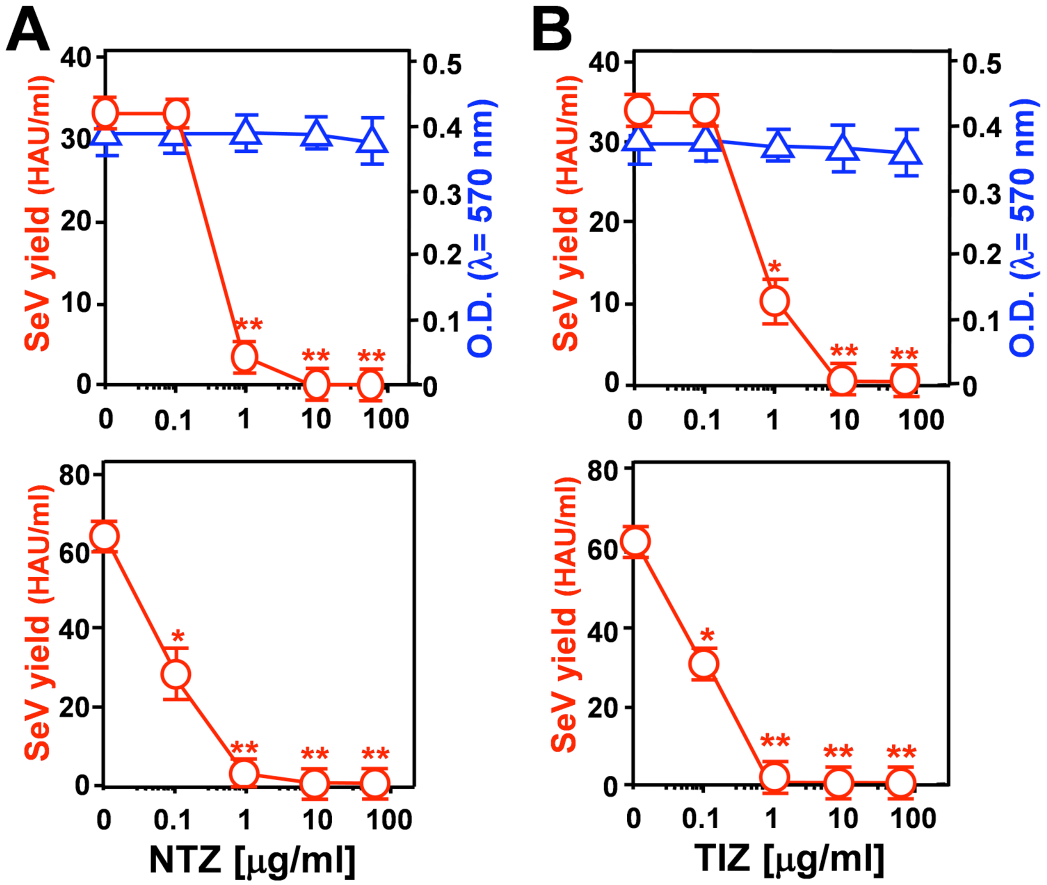

C

Drug	MOI (3 PFU/cell) IC$_{50}$ (µg/ml)	SI	MOI (0.01 PFU/cell) IC$_{50}$ (µg/ml)	SI
Nitazoxanide	0.30 ± 0.01	>167	0.08 ± 0.02	>625
Tizoxanide	0.50 ± 0.01	>100	0.10 ± 0.01	>500

Supplementary Figure 1

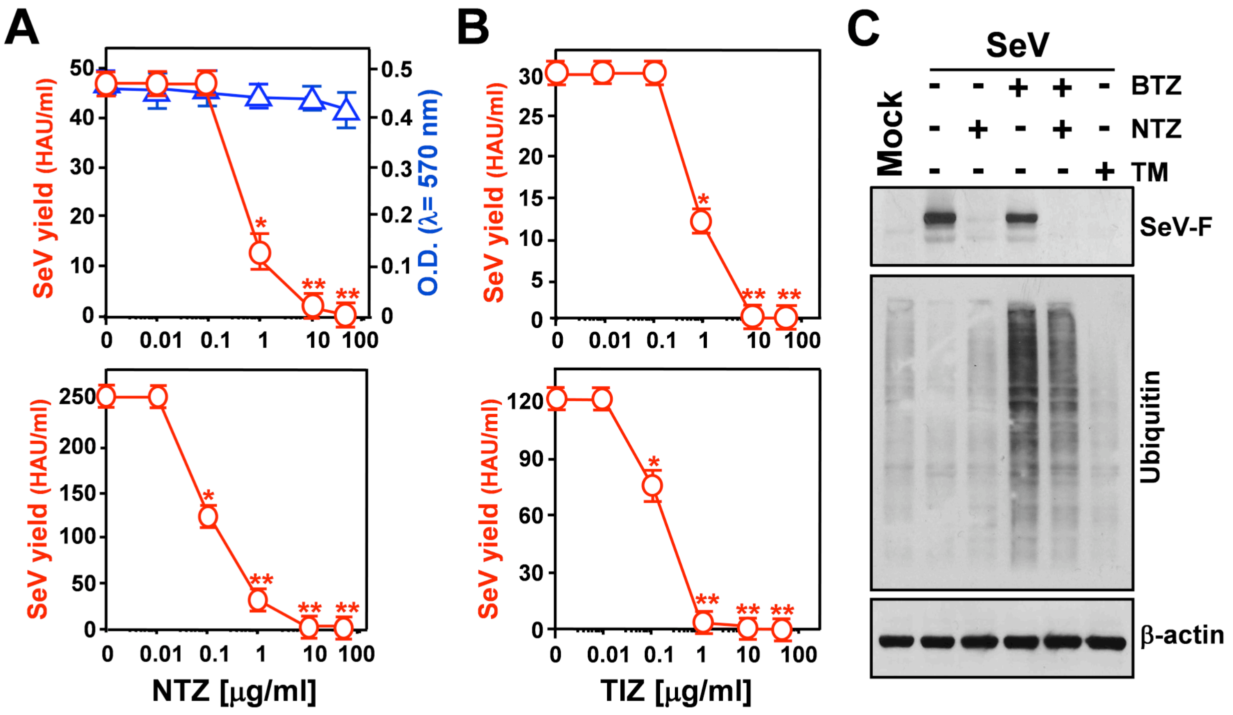

Supplementary Figure 2

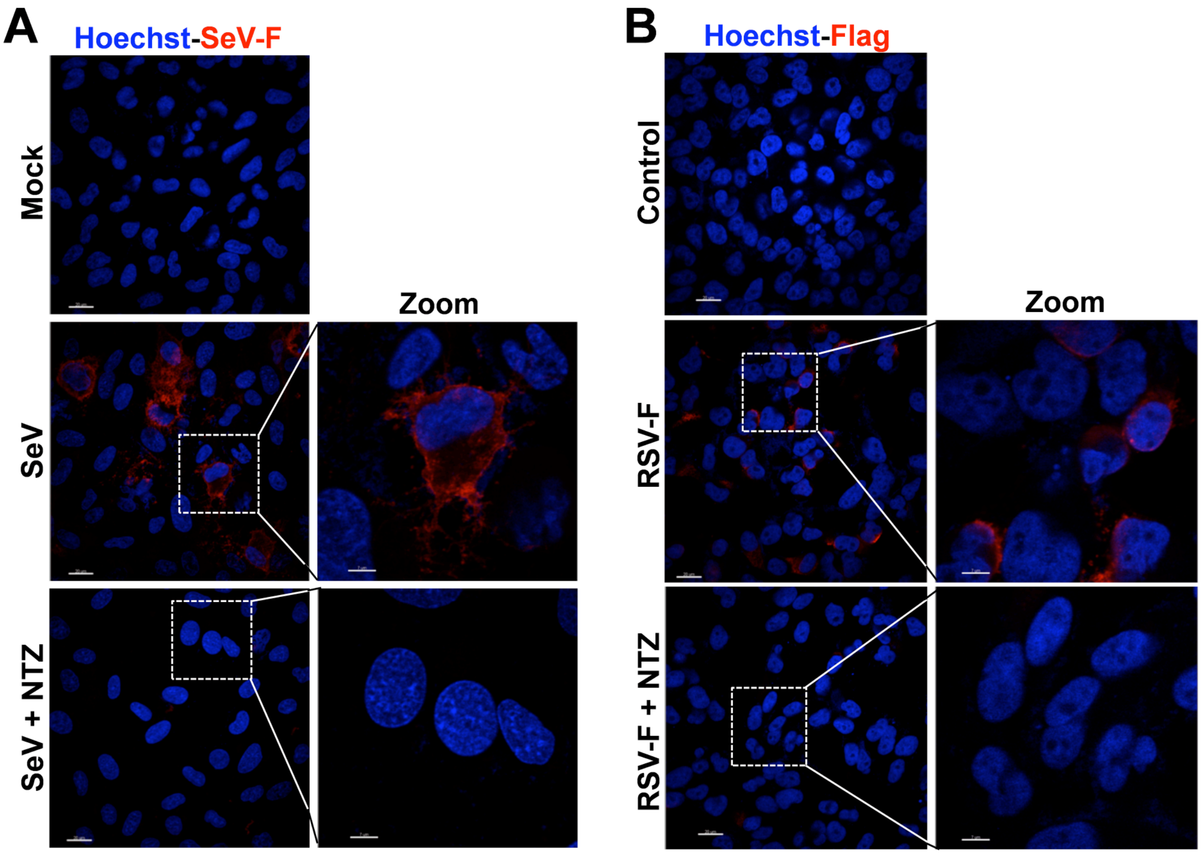

Supplementary Figure 3

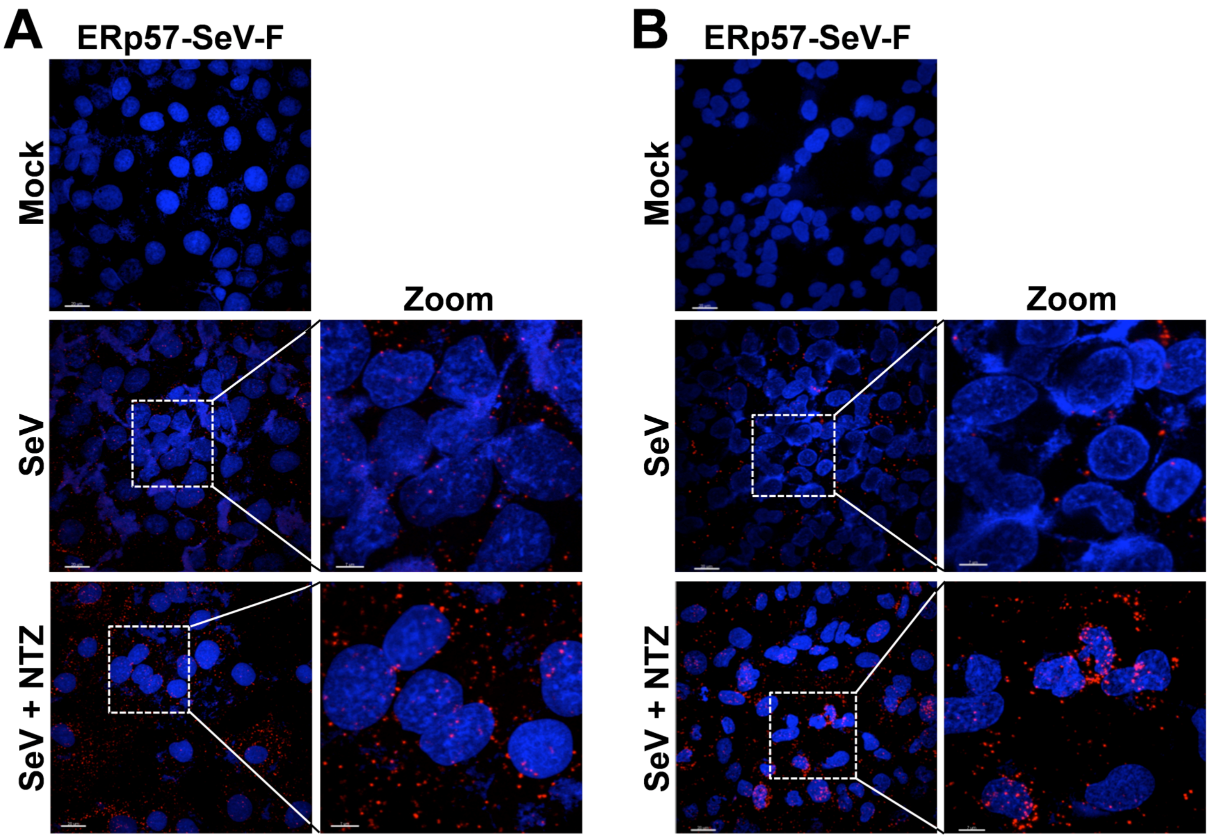

Supplementary Figure 4

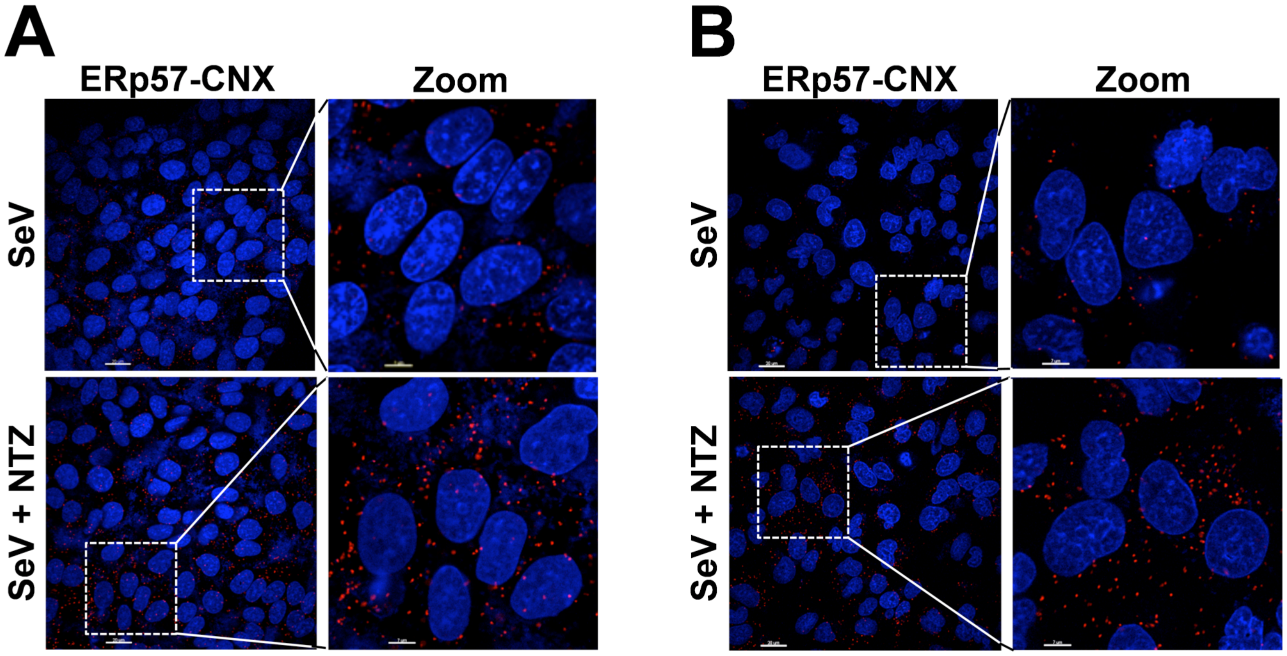

Supplementary Figure 5

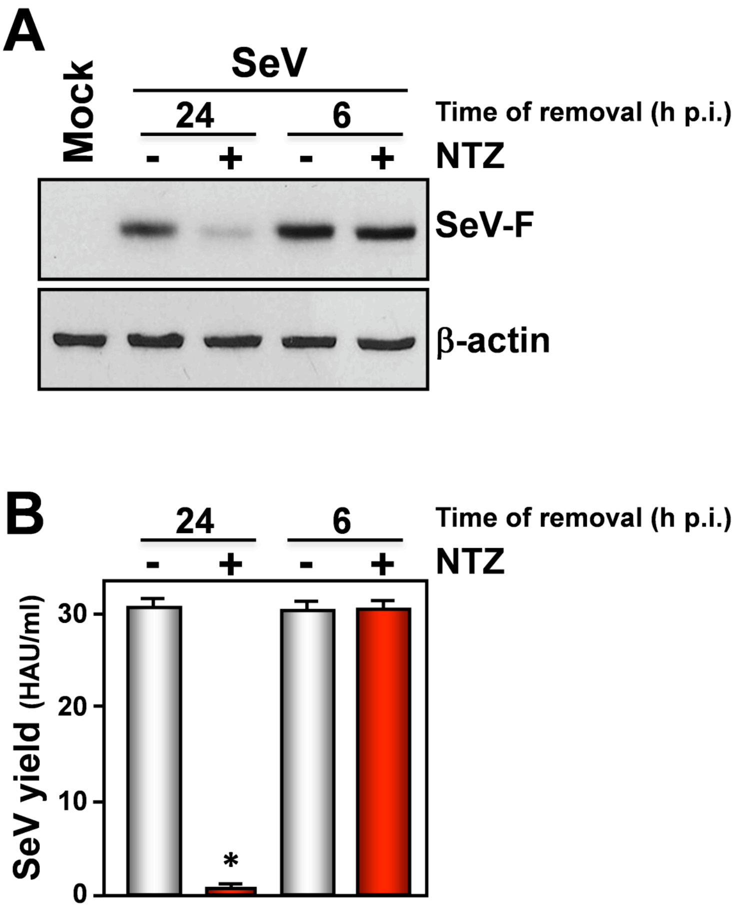

Supplementary Figure 6

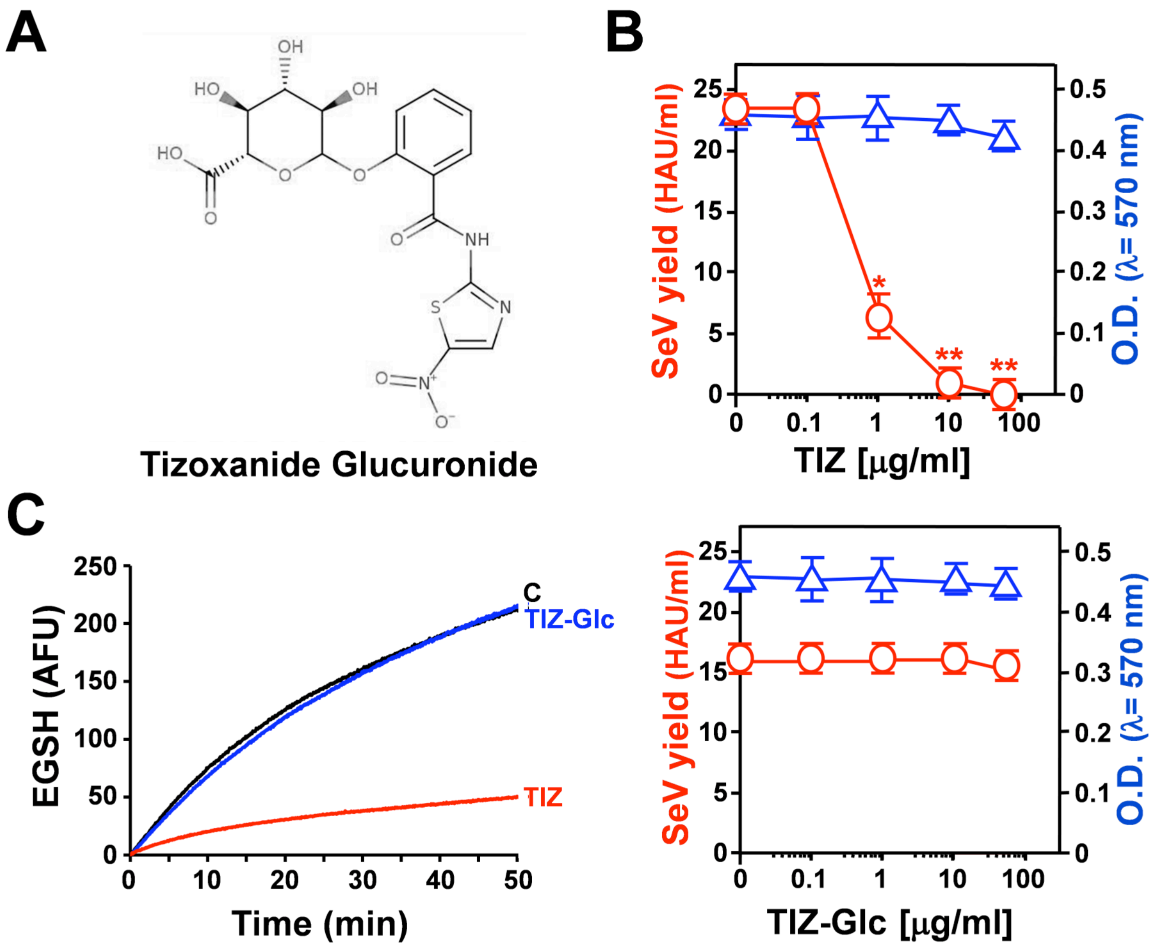

Supplementary Figure 7

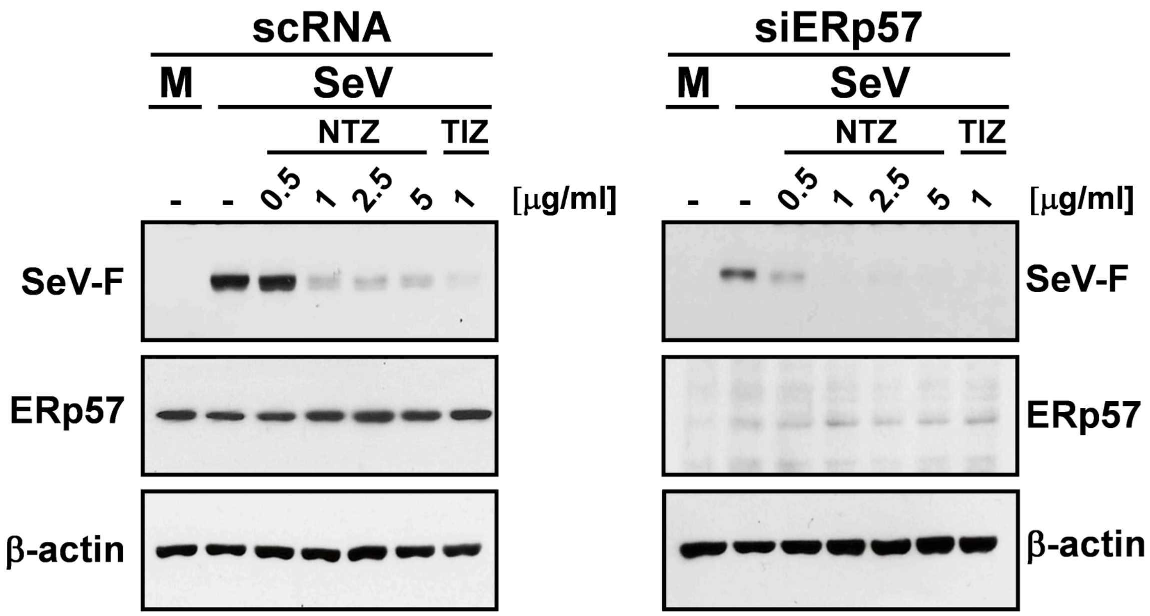

Supplementary Figure 8

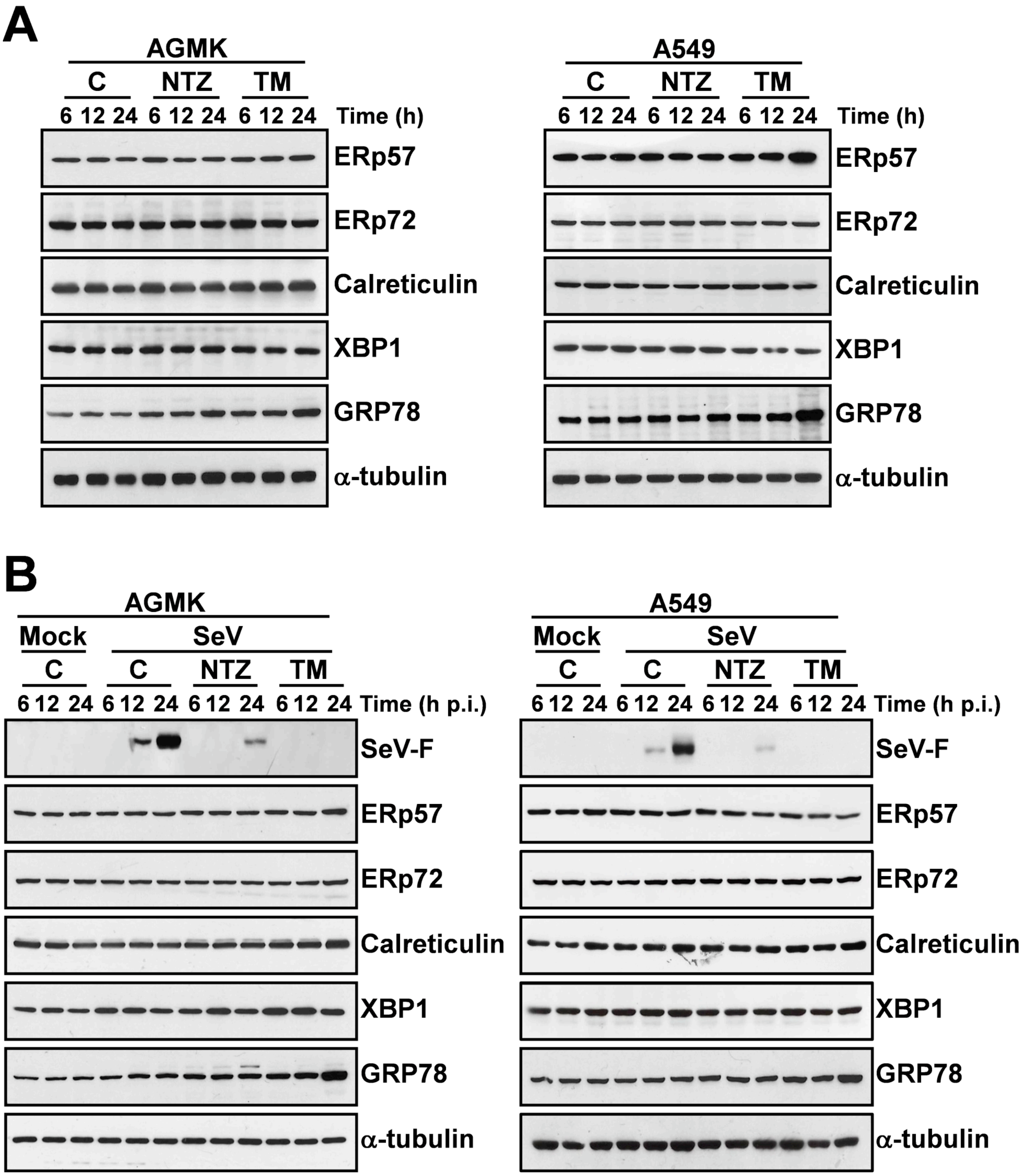

Supplementary Figure 9

Fig. 2-B

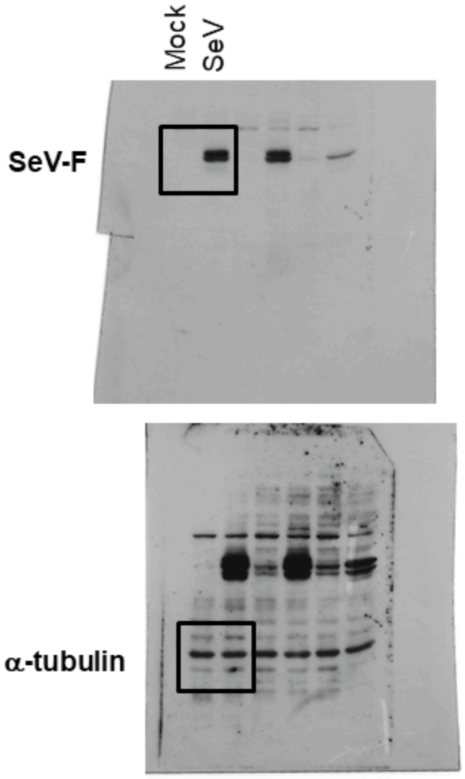

Fig. 2-C

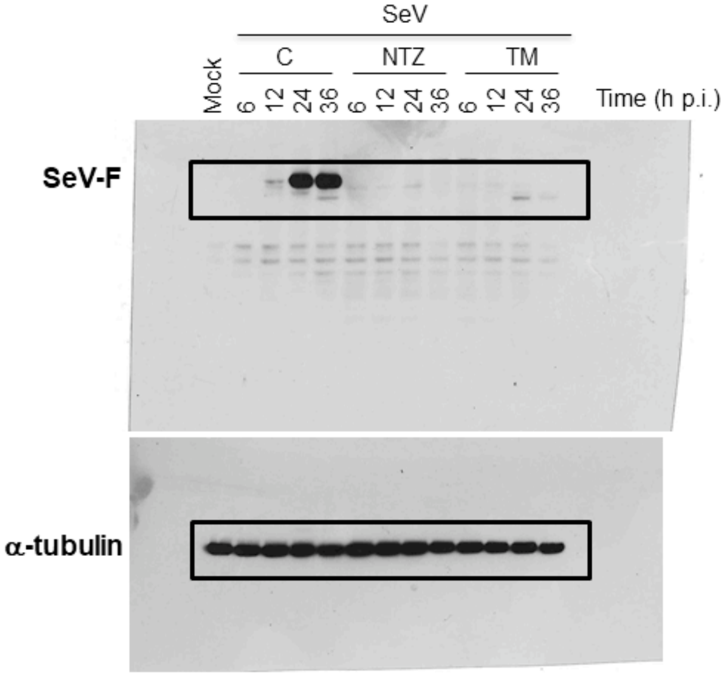

Supplementary Figure 10

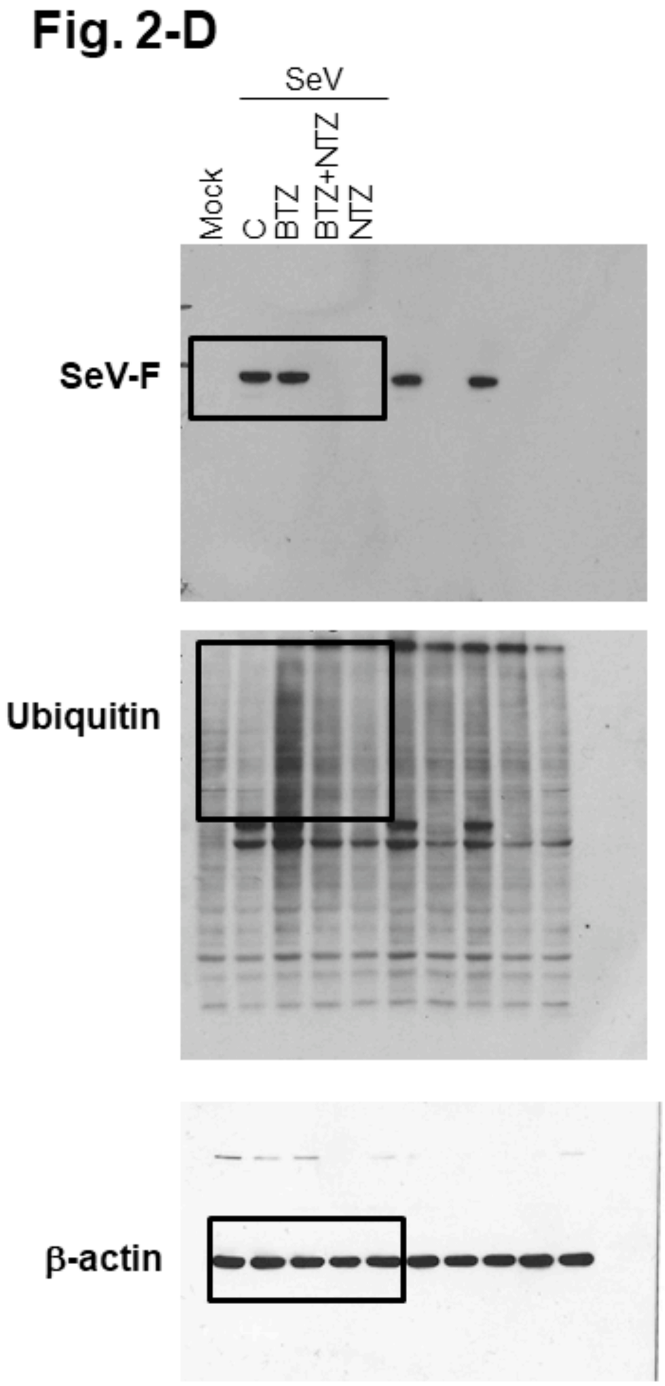

Supplementary Figure 11

Fig. 3-A

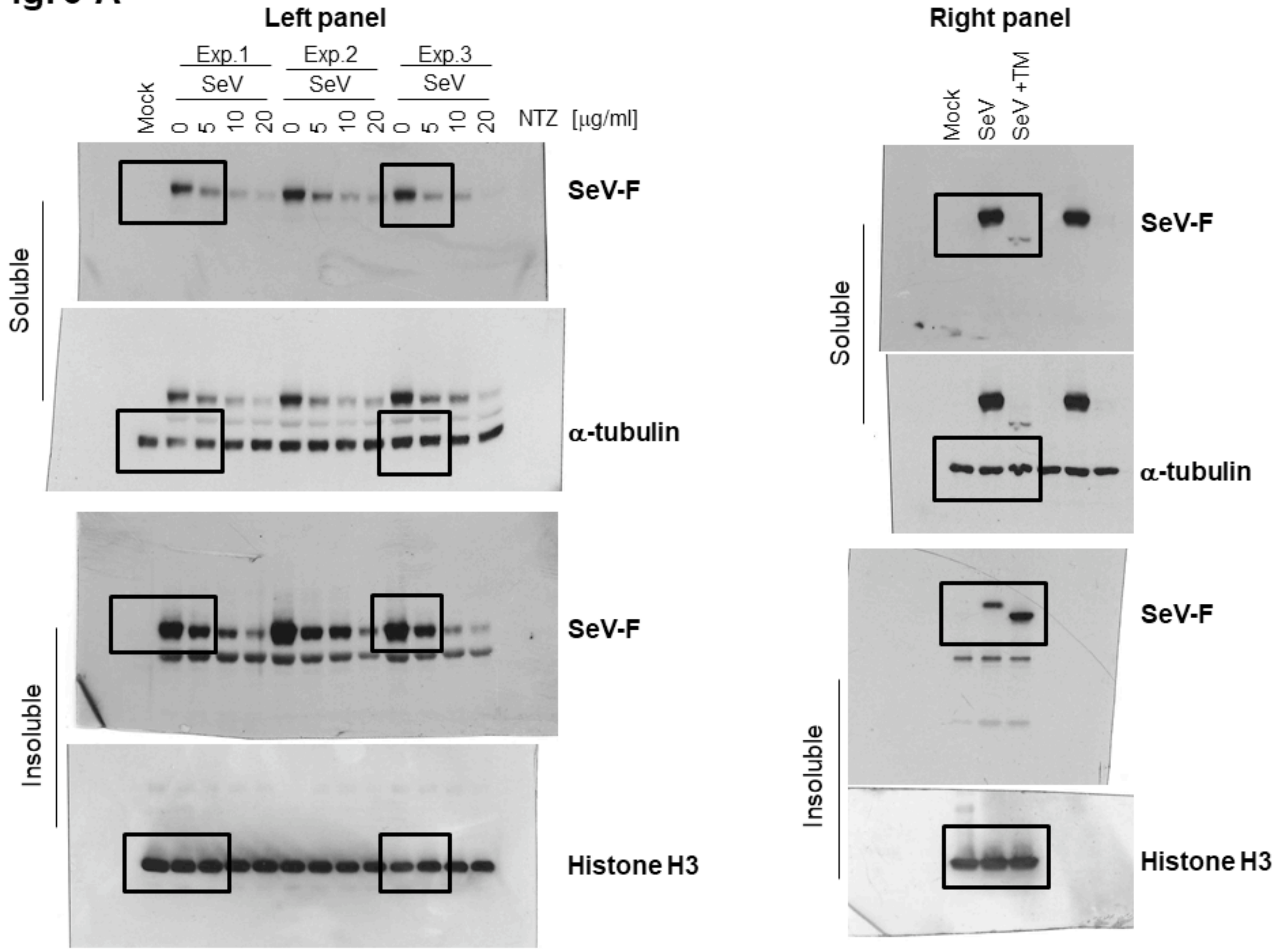

Supplementary Figure 12

Fig. 3-B

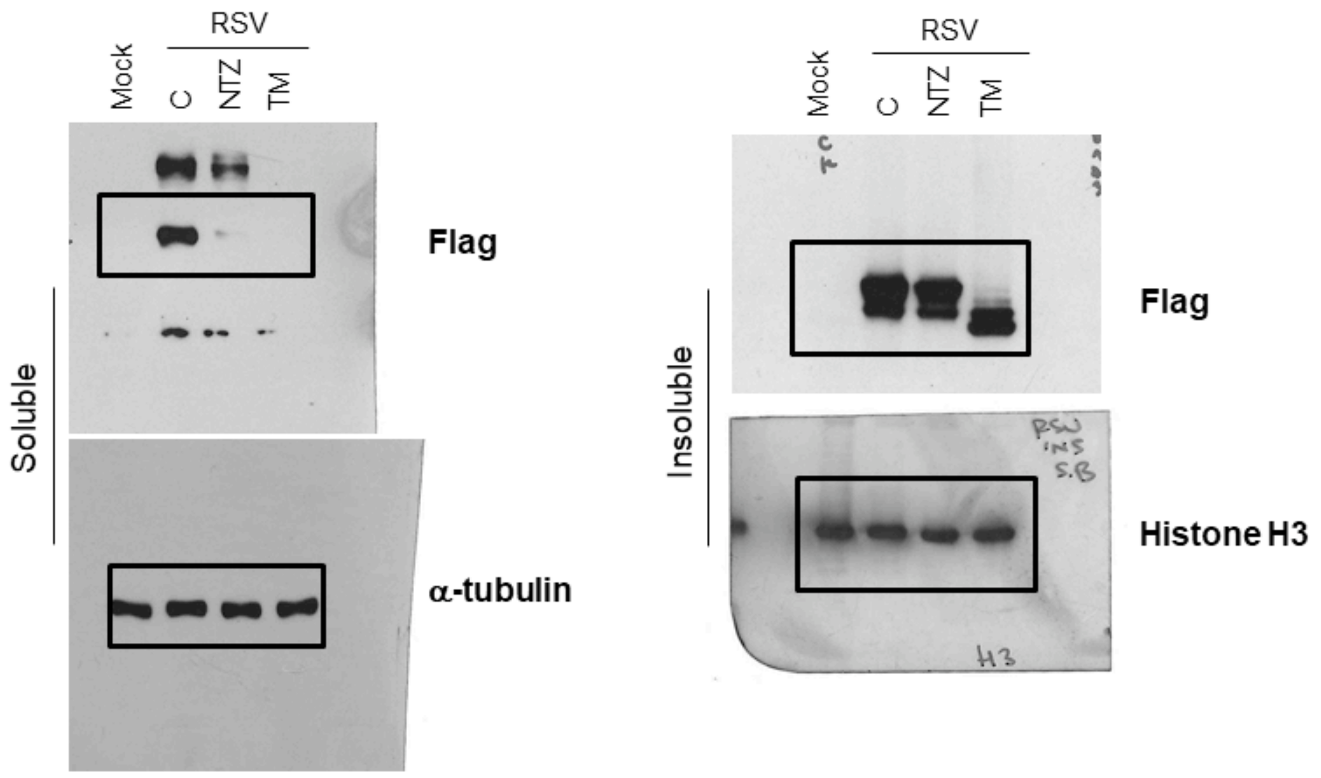

Supplementary Figure 13

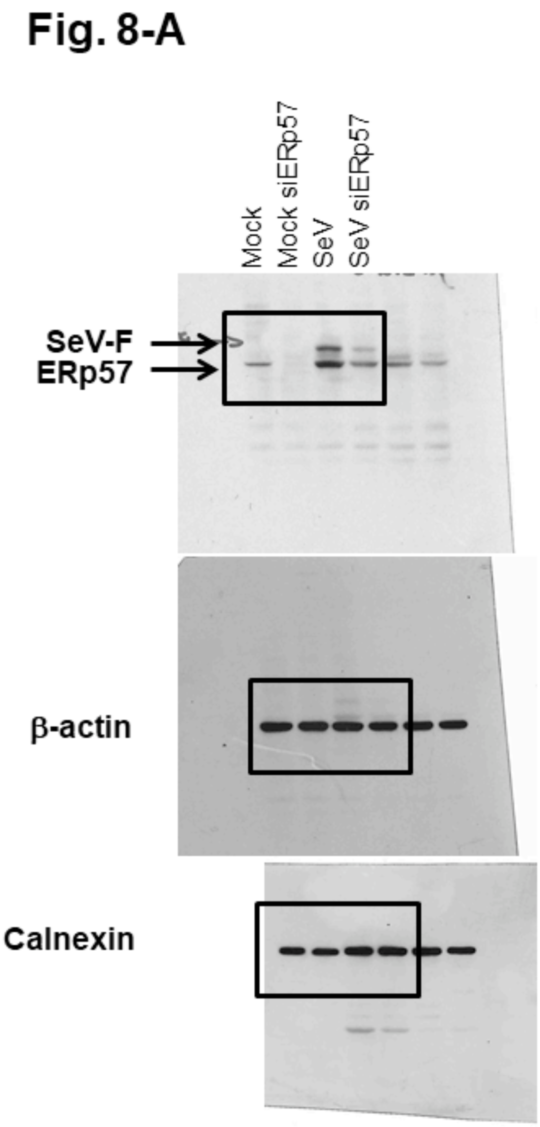

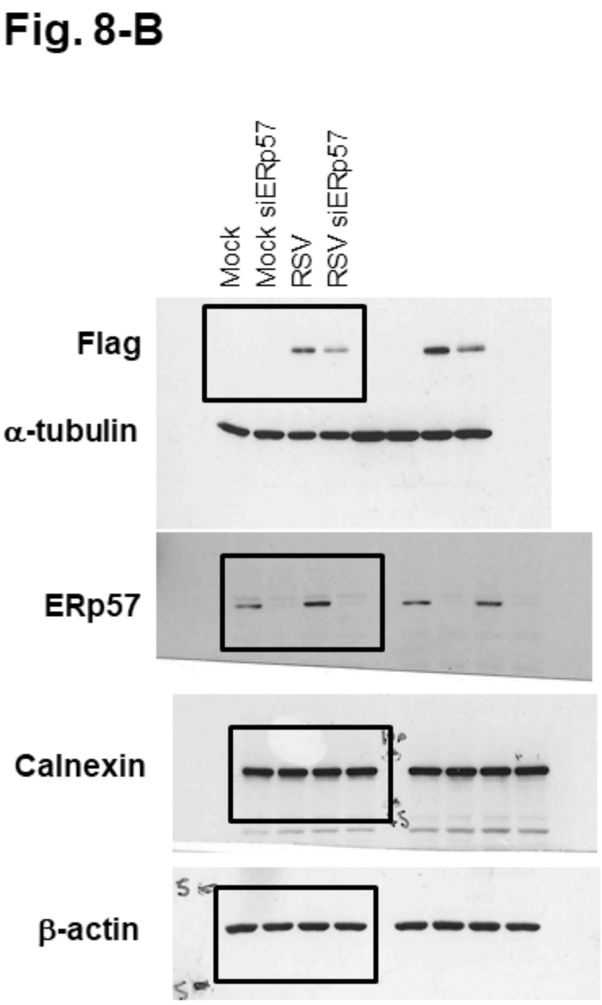

Supplementary Figure 14

Supplementary Fig. 2-C

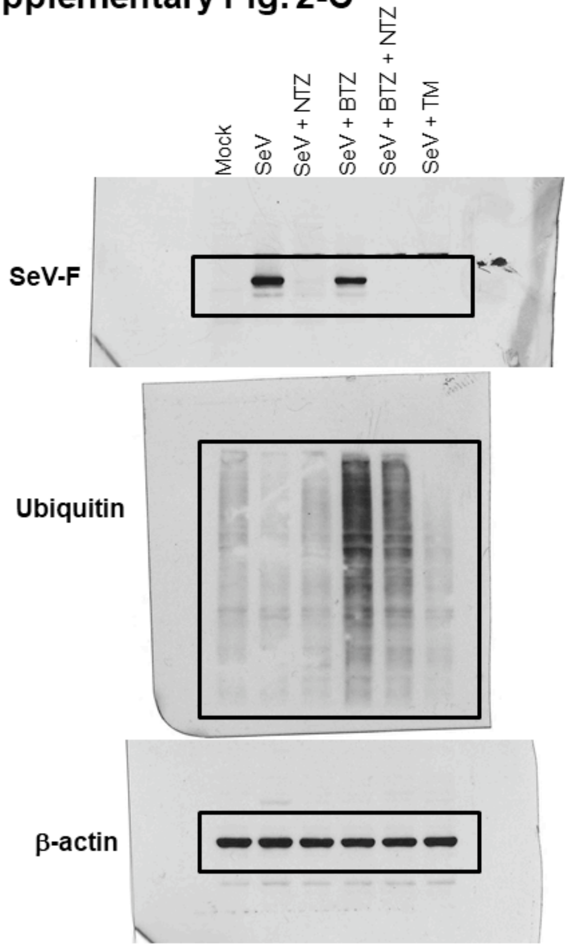

Supplementary Fig. 6-A

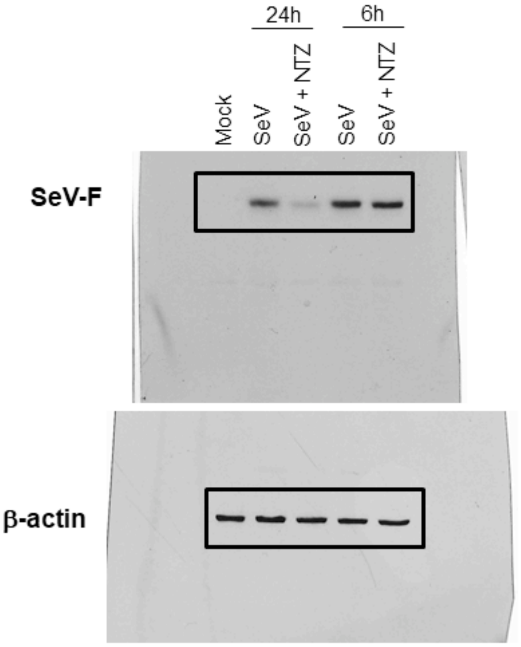

Supplementary Figure 15

Supplementary Fig. 8

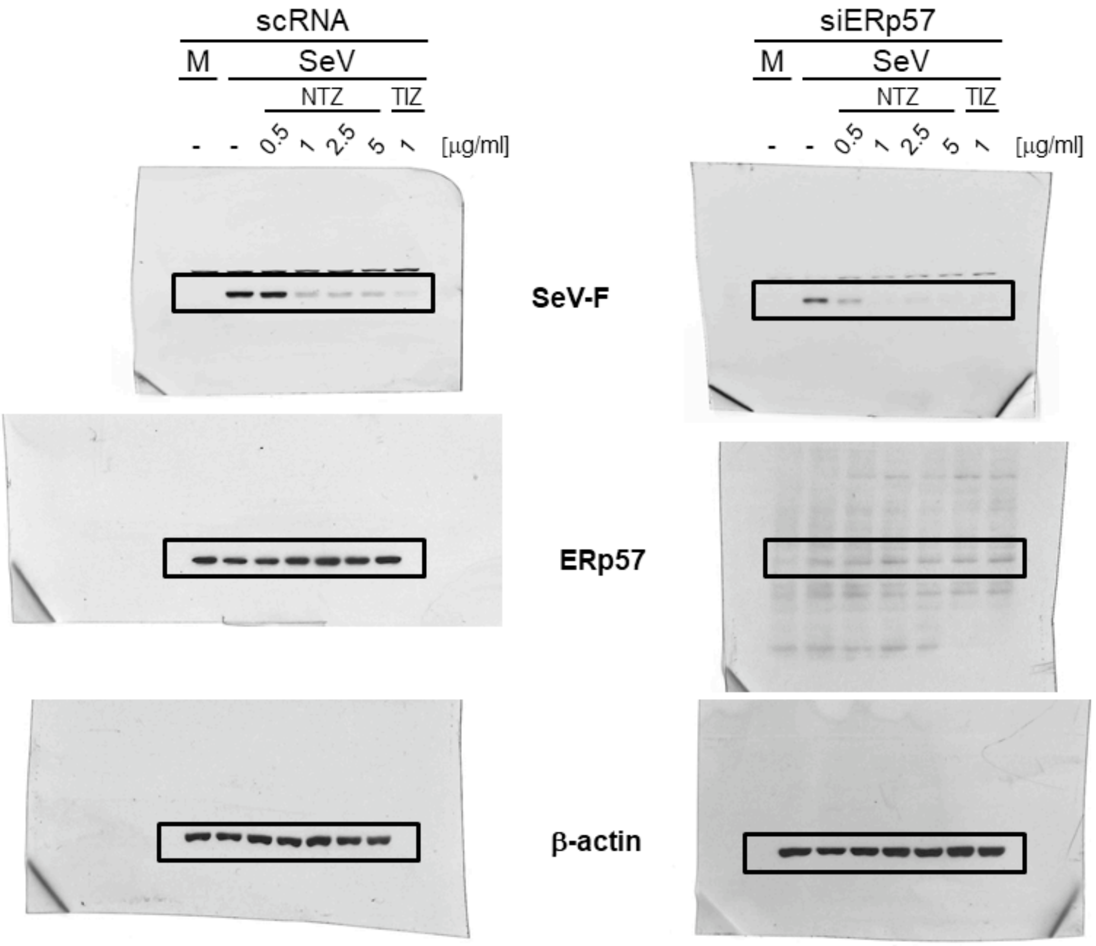

Supplementary Figure 16

Supplementary Fig. 9-A

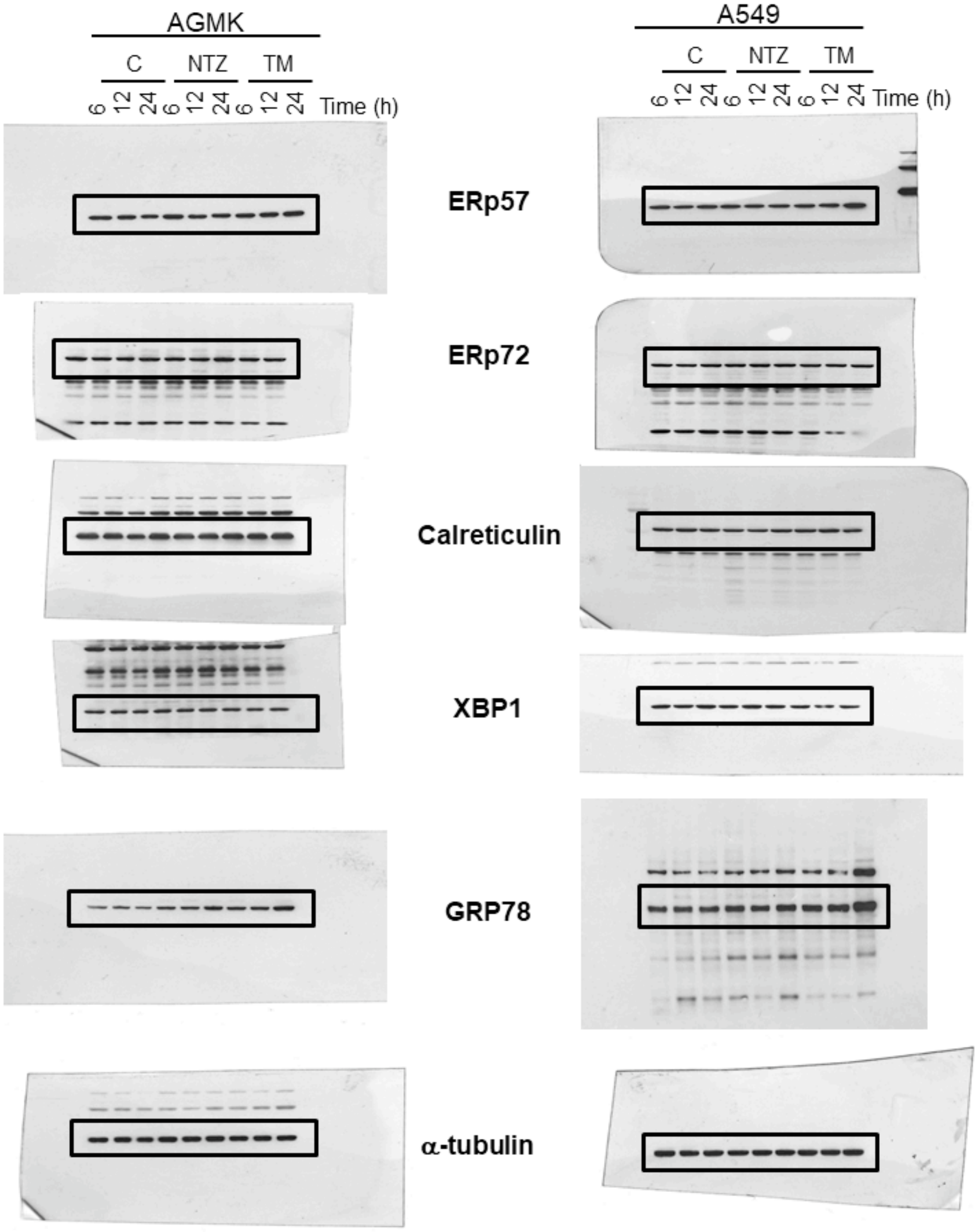

Supplementary Figure 17

Supplementary Fig. 9-B/AGMK

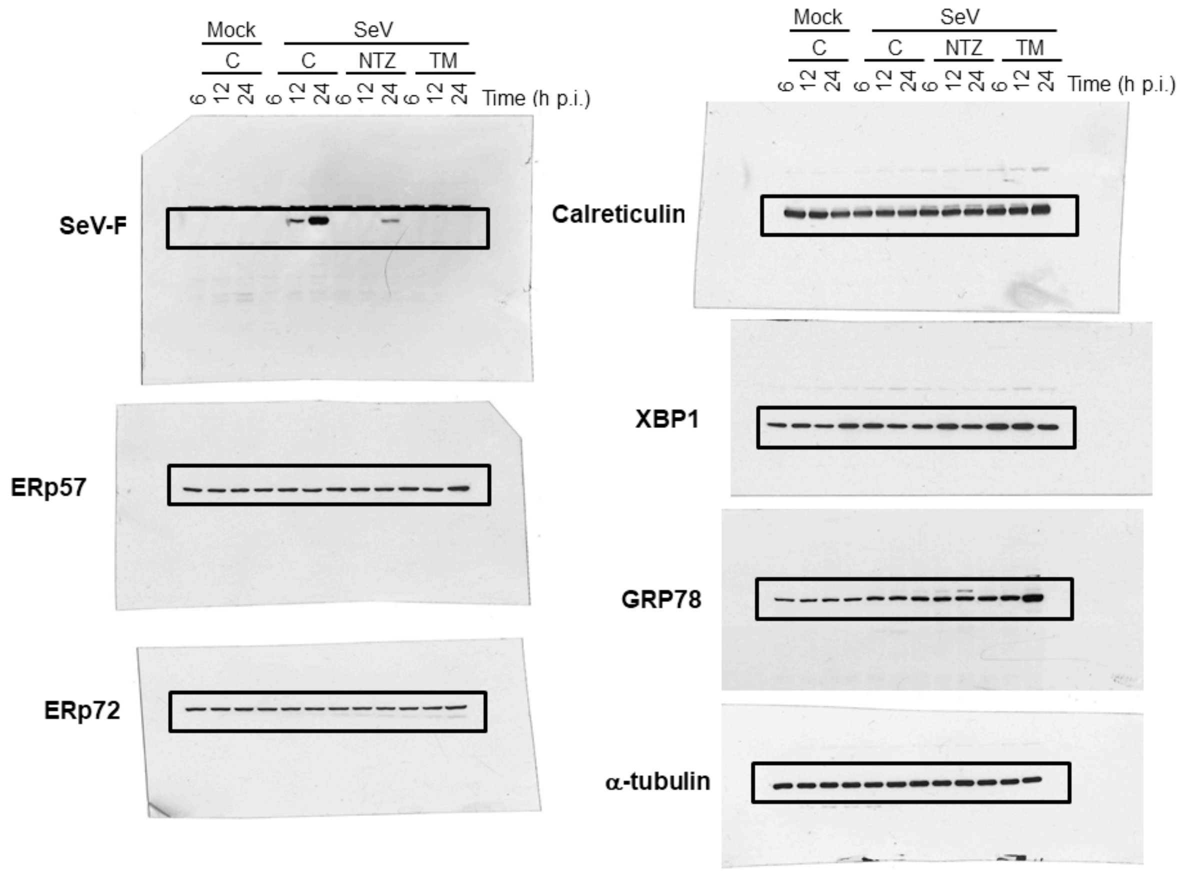

Supplementary Figure 18

Supplementary Fig. 9-B/A549

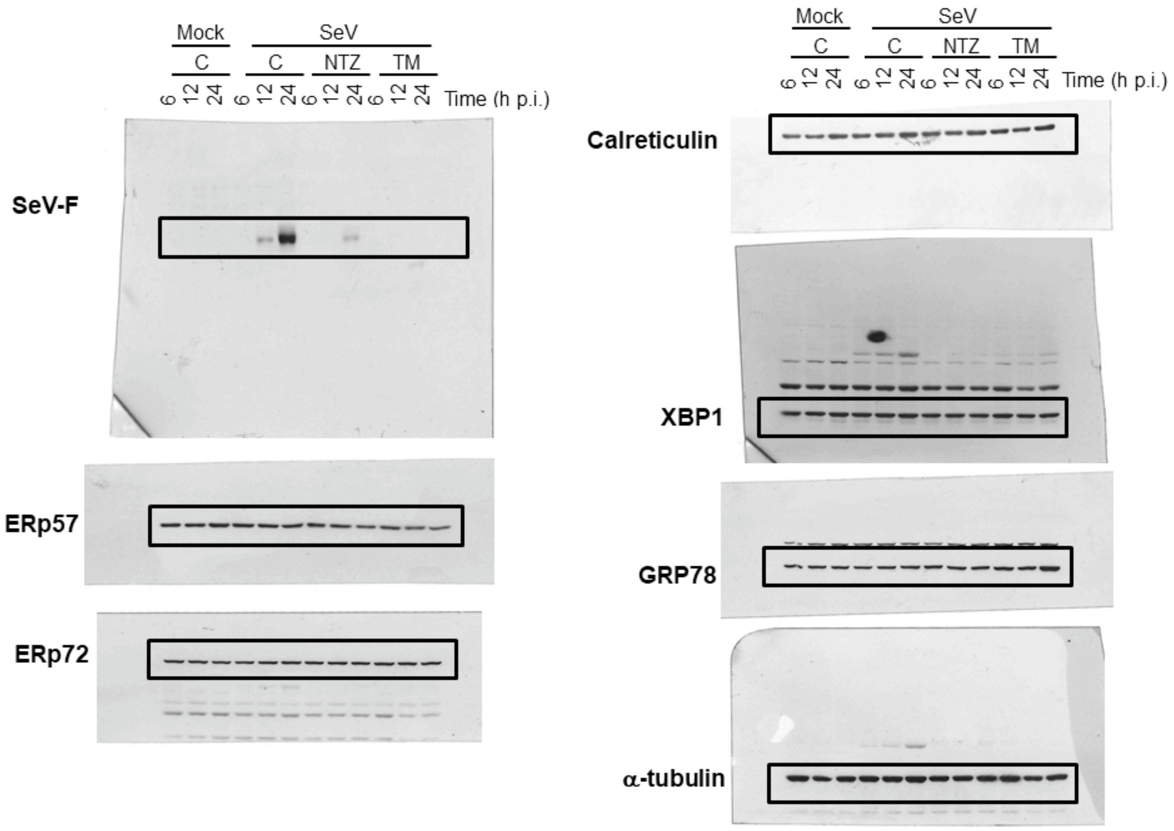

Supplementary Figure 19
